# Structural basis for the evolution of a domesticated group II intron–like reverse transcriptase to function in host cell DNA repair

**DOI:** 10.1073/pnas.2504208122

**Published:** 2025-07-29

**Authors:** Seung Kuk Park, Mo Guo, Jennifer L. Stamos, Wantae Kim, Sidae Lee, Y. Jessie Zhang, Alan M. Lambowitz

**Affiliations:** ^a^Department of Molecular Biosciences, University of Texas at Austin, Austin, TX 78712; ^b^Department of Oncology, Dell Medical School, University of Texas at Austin, Austin, TX 78712

**Keywords:** reverse transcriptase evolution, DNA repair, retroelements, retrotransposons, LINE-1 elements

## Abstract

A previous study found that a bacterial group II intron–like reverse transcriptase (G2L4 RT) evolved to function in double-strand break repair (DSBR) via microhomology-mediated end-joining (MMEJ) and that a mobile group II intron-encoded RT has a basal DSBR activity that uses conserved structural features of non-long terminal repeat (non-LTR)-retroelement RTs. Here, we determined G2L4 RT apoenzyme and snap-back DNA synthesis structures revealing unique structural adaptations that optimized its cellular function in DSBR. These included an RT3a structure that stabilizes the apoenzyme in an inactive conformation until encountering a DNA substrate; a longer N-terminal extension/RT0-loop with conserved residues that together with a modified active site favors strand annealing; and a conserved dimer interface that localizes G2L4 RT homodimers to DSBR sites with both monomers positioned for MMEJ. Our findings reveal how an RT can function in DNA repair and suggest ways of optimizing related RTs for genome engineering applications.

## Significance

Bacteria encode a variety of reverse transcriptases (RTs) that evolved from mobile group II intron RTs to perform different cellular functions. A previous study found that one such domesticated group II intron–like RT (G2L4 RT) functions in double-strand break repair (DSBR) and that a closely related mobile group II intron-encoded RT has a basal DSBR activity. Here, X-ray crystal structures of apoenzyme and DNA substrate-bound G2L4 RT together with biochemical and mutational analyses revealed a series of structural adaptations that optimized its cellular function in DSBR. The structures provide insights into how an RT can function in DNA repair and suggest ways of optimizing RTs for genome engineering applications.

Reverse transcriptases (RTs) are ancient enzymes that evolved from an RNA-dependent RNA polymerase likely during the transition from an RNA to a DNA world (1–4). Although RTs are best known for their association with retroviruses, they remain prevalent in bacteria both as RTs encoded by retrotransposons called mobile group II introns and as chromosomally encoded RTs that evolved from group II intron RTs to perform cellular functions, a process referred to as domestication (5–8). Mobile group II introns are hypothesized to have entered ancestral eukaryotes with bacterial endosymbionts that gave rise to mitochondria and chloroplasts, proliferated in what became the nuclear genome, and ultimately evolved into eukaryotic spliceosomal introns and the core of the eukaryotic RNA splicing apparatus (snRNAs U2, U5, and U6 and spliceosomal protein Prp8), as well as into eukaryotic RTs (9–13). The latter include non-LTR-retrotransposon RTs, a family of enzymes that includes human LINE-1 element RT and whose RT cores remain closely related to those of group II intron RTs (14, 15), followed by more divergent telomerase and retroviral RTs (13).

Genome sequencing revealed that bacteria harbor a wide variety of chromosomally encoded group II intron–like RTs that are no longer associated with group II introns and have distinctive conserved structural features that enable them to perform different cellular functions (5–8). These include phage defense by a variety of mechanisms, including de novo synthesis of new genes, host-phage tropism switching, site-specific integration of RNA protospacers into chromosomal CRISPR arrays, and double-strand break repair (DSBR) via microhomology-mediated end-joining (MMEJ), which was also found to be a basal activity of a group II intron-encoded RTs (16–24). In many cases, these cellular functions are dependent upon novel structural features and biochemical mechanisms that could not have been rationally designed or even imagined based on existing knowledge.

**Fig. 1.**
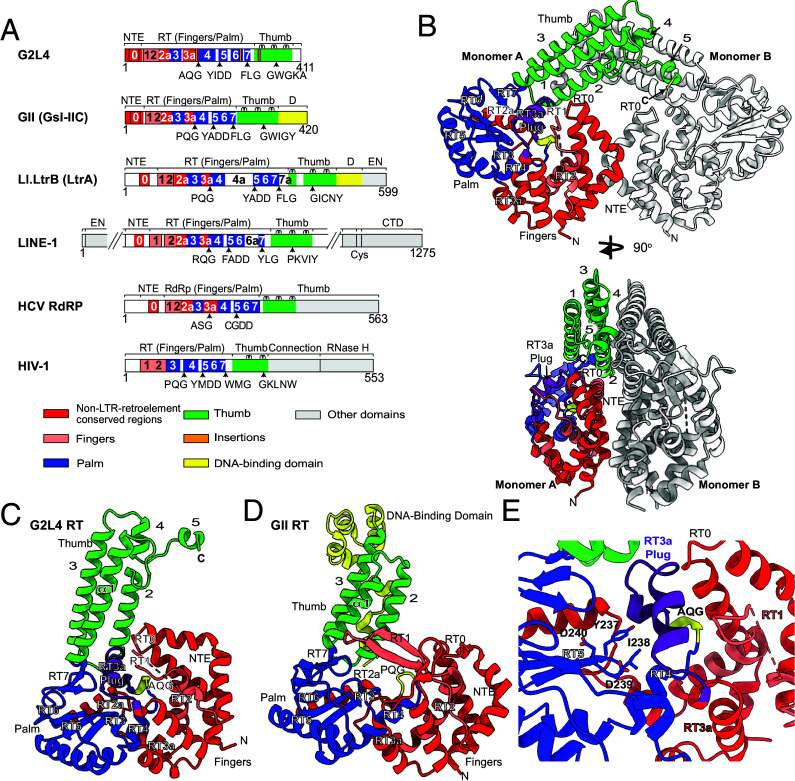
Structure of G2L4 RT apoenzyme. (*A*) Schematics comparing G2L4 RT to Group IIC intron GsI-IIC (denoted GII) RT, Group IIA intron Ll.LtrB (LtrA protein), a human LINE-1 element RT, HCV RdRP, and retrovirus HIV-1 RT. RT1 to 7 are conserved sequence blocks in the fingers (salmon) and palm (blue) of all RTs. An NTE with an RT0 loop, RT2a, and RT3a (red) are additional functionally important regions of non-LTR-retroelement RTs and RdRPs that are absent in retroviral RTs. (*B*) G2L4 RT apoenzyme crystal structure (90°-rotated views) with monomer A fingers, palm, and thumb colored as in panel *A*, AQG motif yellow, disordered RT1 salmon-colored dashed line, and monomer B gray. N and C, N-, and C-termini, respectively. (*C*) Structure of a G2L4 RT apoenzyme monomer. (*D*) Structure of GII RT in active conformation (PDB: 6AR1) (4), with bound template/primer. (*E*) G2L4 RT active-site region in the apoenzyme structure blocked by the RT3a plug (purple) with active site YIDD residues shown as blue sticks with oxygens red.

All RTs share a common structural framework composed of fingers, palm, and thumb or thumb surrogate regions that fold into a hand-like structure, forming a cleft containing an RT active site composed of three conserved aspartate residues that bind catalytic Mg^2+^ ions (1, 25–27). The RT fingers and palm contain seven conserved sequence blocks (RT1-7) that include or are positioned around the RT active site to contribute to RT activity (Fig. 1*A*) (26, 27). Bacterial and other prokaryotic RTs, mitochondrial retroplasmid RTs, and eukaryotic non-LTR-retrotransposon RTs, collectively termed non-LTR-retroelement RTs, contain three additional regions: an N-terminal extension (NTE) with an RT0 loop and two expanded regions (RT2a and RT3a) between conserved RT sequence blocks. In group II intron RTs, these regions contribute to tighter binding pockets that enable higher fidelity and processivity than retroviral RTs (4, 28). Structural cognates of the NTE/RT0 loop, RT2a and RT3a, are also present and functionally important in RNA-dependent RNA polymerases (RdRPs), which were evolutionary ancestors of RTs, but they have been lost from retroviral RTs, which evolved a looser architecture and more promiscuous lifestyle to introduce and propagate mutational variations that help retroviruses evade host defense (Fig. 1*A*) (4, 28).

In a previous study, we showed via genetic and biochemical analyses that a *Pseudomonas aeruginosa* group II intron–like (G2L4) RT functions in DSBR via MMEJ both in its native host and when transferred into *Escherichia coli* (21). Here, we determined a 2.6-Å crystal structure of a full-length G2L4 RT apoenzyme and a 2.8-Å cocrystal structure of G2L4 RT bound in an active conformation to a snapback DNA synthesis substrate and incoming dNTP. The structures revealed a series of structural adaptations that evolved to optimize the DSBR function of G2L4. Our findings enabled a detailed structural model of how G2L4 RT functions in DSBR and draw attention to the NTE/RT0 loop, RT2a, and RT3a as regions that can be modified to optimize non-LTR-retroelement RTs for biotechnological applications.

## Results

### G2L4 RT Apoenzyme Structure.

Group II intron-encoded RTs typically bind group II intron RNAs cotranscriptionally to promote RNA splicing and tend to be unstable when removed from the intron RNA (29, 30). By contrast, domesticated group II intron–like RTs are synthesized as free-standing proteins that must bind their physiological substrates at different intracellular locations while avoiding deleterious interactions with cellular RNAs. To investigate how this might be done by G2L4 RT, we obtained a crystal structure of the full-length G2L4 RT apoenzyme (Fig. 1*B*). The structure was solved at 2.60-Å resolution in space group P2_1_ by de novo phasing using selenomethionine (*SI Appendix*, Table S1). Unlike group II intron RTs, which typically appear in crystal or cryo-EM structures as monomers (4, 31–36), the G2L4 RT apoenzyme structure revealed two monomers per asymmetric unit forming a symmetric dimer, a difference verified by size-exclusion chromatography comparing G2L4 RT to a closely related mobile group II intron RT (*Geobacillus stearothermophilus* group IIC intron RT, denoted GII RT; *SI Appendix*, Fig. S1*A*). Continuous density could be traced for all amino acids, except those in two putative loops (Q65 to R78 in RT1 and G116 to D120 in RT2a; Fig. 1 *B* and *C*).

The two G2L4 RT monomers that form the apoenzyme dimer have nearly identical hand-like folds composed of fingers, palm, and thumb regions (RMSD = 0.83 Å for main chain carbon atoms; *SI Appendix*, Fig. S1*B*), but lack DNA-binding (D) or DNA endonuclease (En) domains found in group II intron RTs (Fig. 1 *A*–*D*). Although G2L4 RT has relatively low overall sequence identity to GII RT (EMBOSS Needle sequence identity = 21.5%; *SI Appendix*, Fig. S1*C*), its tertiary structure is similar to that of GII RT (Fig. 1 *C* and *D*).

The fingers and palm of G2L4 RT contain conserved sequence blocks RT1-7 plus an NTE with an RT0 loop and RT2a and RT3a between the conserved sequence blocks (Fig. 1*A* and *SI Appendix*, Fig. S1*C*). The thumb domain of G2L4 RT is similar to that of GII RT in being composed of three parallel α-helices (α1-3), but differs in extending straight up for 41 Å, 15 Å farther than in GII RT, and ending with additional short α-helices (α4 and α5) that interact with the thumb domain of the opposite monomer (Fig. 1 *B*–*D*) (4).

The structures of most of the conserved RT motifs and the active site of G2L4 RT apoenzyme are similar to those of GII RT in an active conformation with bound substrates (4). Two exceptions were i) RT1, which ordinarily forms two antiparallel β-strands including a fingertips loop over the RT active site, but was disordered and thus not visible in the G2L4 apoenzyme structure (represented by a salmon dashed line in Fig. 1*C*), and ii) the XQG motif (AQG in G2L4, PQG in GII RT), a critical element of the RT active site that functions in dNTP binding (4), but is folded inward in the G2L4 RT apoenzyme structure compared to the active conformation of GII RT (Fig. 1 *C* and *D*).

As in GII RT, the NTE of the G2L4 RT apoenzyme is composed of two bent α-helices separated by a sharp turn that forms an RT0 loop, but with the RT0 loop smaller and the bent α-helices longer and closer to the RT active site than in the active structure of GII RT (Fig. 1 *C* and *D* and *SI Appendix*, Fig. S1*C*). RT2a is nearly identical to that in the GII RT cocrystal structure, but with a hinge region (G116 to D120) that connects to RT2 and is disordered in the absence of bound substrate (Fig. 1 *C* and *D*). By contrast, RT3a in G2L4 RT differs markedly from that in group II intron RTs in forming a novel structural feature, a double-helical plug (denoted RT3a plug; highlighted in purple in Fig. 1 *C* and *E*). The RT3a plug is a structural adaptation that completely blocks the active site of G2L4 RT, rendering the apoenzyme inactive until it encounters a physiological substrate, as described further below.

### Interaction of the RT3a Plug with the G2L4 RT Active Site.

**Fig. 2.**
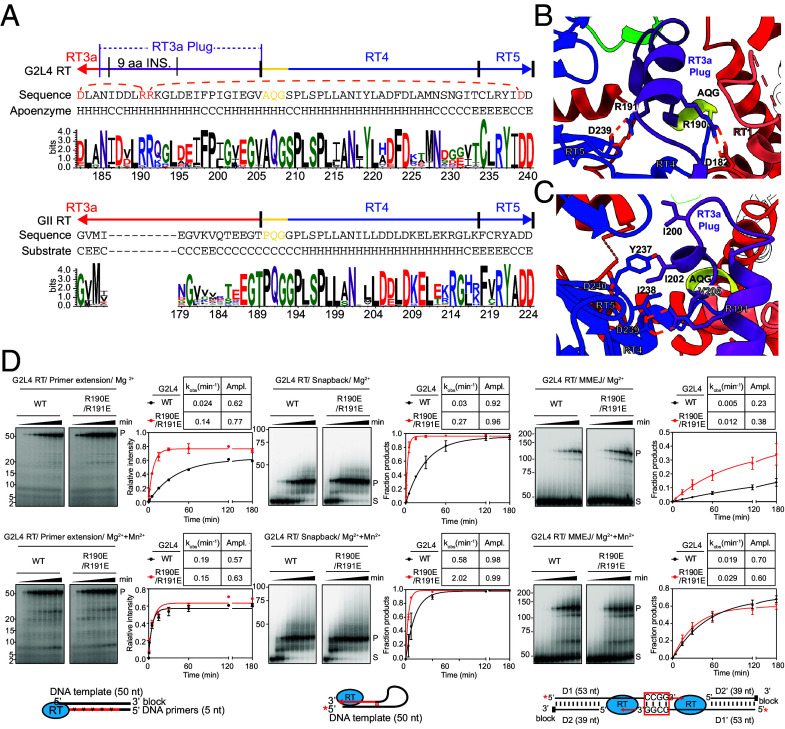
Characteristics of the G2L4 RT RT3a plug. (*A*) WebLogos comparing conservation of amino acid residues in the C-terminal region of RT3a, RT4, and RT5 of G2L4 and GII RTs. WebLogos were based on 130 G2L4 RTs and 500 GsI-IIC RTs identified by BLASTP as having ≥50% amino acid sequence identity aligned by ClustalW (http://www.clustal.org/clustal2/). Amino acid residues in α-helical (H), random coil (C), and β-sheet (E) regions are shown below the amino acid sequence. Interacting residues in G2L4 RT described in the text are highlighted in red and connected by orange dashed lines. (*B*) Salt bridges between conserved residues in the RT3a plug and active site of G2L4 RT apoenzyme. Interacting residues R190 and R191 in the RT3a plug and D182 and D239 in RT3a and RT5, respectively, are shown as sticks with carbons colored by region and nitrogens and oxygens blue and red, respectively. Electrostatic salt bridges are depicted by orange dashed lines. (*C*) Right-hand rotated view of panel *B*, showing the interactions of the RT3a plug (purple) with the YIDD motif at the G2L4 RT active site. (*D*) Biochemical activities of WT and RT3a mutant MBP-tagged G2L4 RT in reaction media containing 10 mM Mg^2+^ (*Top* row) or 10 mM Mg^2+^ plus 1 mM Mn^2+^ (*Bottom* row). *Left* panels, primer extension assays with a 50-nt 3’-inverted dT-blocked DNA template, a 5-nt DNA primer, and 1 mM dNTPs plus trace ^32^P-labeled dTTP; middle panels, snapback DNA synthesis assays with a 5’-^32^P-labeled (red asterisk) 50-nt DNA substrate; *Right* panels, MMEJ assays with a 5’-^32^P-labeled (red asterisk) 53-nt preannealed partially double-stranded DNAs with an inverted dT 3’-blocking group on one strand (D2 and D2’) and 3’ overhangs with complementary 3’-CCGG sequences on the opposite strand (D1 and D1’). Reactions were done at 37 °C as time courses up to 180 min, and products (P) and substrates (S) were analyzed in a denaturing polyacrylamide gel for primer extension assays and in a nondenaturing polyacrylamide gel for snapback DNA synthesis and MMEJ assays against ^32^P-labeled synthetic oligonucleotide size markers in a parallel lane (positions indicated to the left of the gel). The plots show the fraction of product based on the relative intensity of product and substrate bands as a function of time, with tables indicating rate constants (k_obs_) and amplitudes (Ampl.) for the production of products with curves fit to a first-order rate equation to obtain average values and variance indicated by error bars for two repeats of the experiment.

The RT3a plug is composed of 21 amino acids (aa) at the distal end of RT3a extending from N185 to V205 just before the conserved AQG of the RT4 motif, and it includes a 9-aa insertion that is conserved in G2L4 RTs but is absent in group II intron or other non-LTR-retroelement RTs (Fig. 2*A* and *SI Appendix*, Fig. S2*A*). These 21 amino acids form two α-helices that plug into the RT active site flush against the conserved YIDD motif (Figs. 1*E* and 2 *B* and *C*), replacing a bound catalytic Mg^2+^ ion at this location in the active structures of GII and other RTs (4, 15, 37). The RT3a plug was not predicted by any folding prediction program, including the most recent version of AlphaFold (AlphaFold 3.0, https://alphafoldserver.com/), indicating that it was beyond the imagination of AI. A strong interaction of the RT3a plug with the G2L4 RT active site is likely what displaced the RT1 β-strands that normally occupy this region causing them to become disordered and pulled the adjacent AQG motif inward where it became inaccessible to bulk solvent, as judged by modeled accessibility of a hypothetical water molecule.

The closest resemblance to the interaction the RT3a of G2L4 RT with the active site was seen in apoenzyme structures of *Roseburia intestinalis* and *Eubacterium rectale* group II intron RT fingers and palm regions obtained with crystallographic constructs lacking a thumb domain (PDB code: 5HHJ and 5HHL) (38). In both of those structures, the distal end of the shorter RT3a, which lacks the 9-aa insertion, forms a one and a half turn α-helix that partially covers the active site YADD motif, but in an orientation that differs from that in G2L4 RT and does not extend to displace the RT1 β-strands, which remain well-ordered over the active-site pocket (*SI Appendix*, Fig. S2 *B* and *C*) (38). Thus, the 9-aa insertion is a unique evolutionary adaptation that enables complete blockage of the RT active site by RT3a in G2L4 RTs.

The structure of the RT3a plug and its binding in the active-site pocket of G2L4 RT are stabilized by an interaction network that includes salt bridges formed by two conserved arginine residues (R190 and R191) that are located within the 9-aa insertion and form part of the first α-helix of the RT3a plug (Fig. 2 *A*–*C*). The first salt bridge is between R190 of the 9-aa insertion and D182 located upstream in RT3a (Fig. 2*B*). Notably, D182 is a conserved acidic residue in RT3a of G2L4 RTs, but a conserved glycine (G175) in RT3a of GII RTs (Fig. 2*A*), indicating a functionally important covariation that stabilizes the RT3a plug. The second salt bridge is between R191 of the 9-aa insertion and the conserved D239 of the YIDD motif at the active site (Fig. 2 *B* and *C*). This salt bridge may help lock the RT3a plug into the active site, effectively blocking access to the catalytic center. This occupation prevents binding of an incoming dNTP and coordination of a catalytic Mg^2+^ ion at the corresponding aspartate residue, as observed in the crystal structure of GII (4) (*SI Appendix*, Fig. S3 *A* and *B*) and other RTs (15, 37), thereby rendering G2L4 RT inactive.

The interactions of the second α-helix of the RT3a plug with the G2L4 RT active site are largely hydrophobic (*SI Appendix*, Fig. S3 *C* and *D*). These interactions involve a series of greasy residues (I200, I202, V205) on a face of the helix that packs along a hydrophobic tunnel formed by Y237 and I238 of the YIDD motif along with F358 and F360 of the thumb and V123 of RT2a (*SI Appendix*, Fig. S3 *C* and *D*). The contribution of I238 of the YIDD motif to the hydrophobic tunnel potentially results in a more stable interaction with the RT3a plug than an A residue at this position. These hydrophobic interactions also suggest why G2L4 RT has higher primer extension activity at lower salt concentrations (21), which weaken hydrophobic interactions, making it easier for G2L4 RT to transition from an inactive to an active conformation upon binding a nucleic acid substrate.

### The RT3a Plug Stabilizes G2L4 RT and Regulates Its Enzymatic Activity.

To investigate the function of the RT3a plug, we tested the effect of different combinations of three mutations (R190E/R191E, R190E/I202E, and R191E/I202E) in residues of the RT3a plug that were suggested by the apoenzyme structure to stabilize its interaction with the G2L4 RT active site (Fig. 2*A* and *SI Appendix*, Fig. S2 *A* and *B*). For these experiments, wild-type (WT) and mutant G2L4 RTs were expressed with an N-terminal maltose-binding protein (MBP) tag that stabilizes the protein without interfering with biochemical activities (30). All three mutant proteins were expressed at the same level as the WT protein, and their gel filtration profiles showed a similar dimer peak (*SI Appendix*, Fig. S4 *A* and *B*). Differential scanning fluorimetry (DSF) indicated that all three mutant proteins were stably folded, but had lower melting temperatures than the WT protein (*SI Appendix*, Fig. S4*C*), indicating that RT3a interactions stabilize the G2L4 RT apoenzyme.

To explore how the RT3a plug affects the enzymatic activities of G2L4 RT, we compared the primer extension, snap-back DNA synthesis, MMEJ, and terminal transferase activities of WT G2L4 RT and the three RT3a mutants (Fig. 2*D* for mutant 190E/R191E and additional assays, including terminal transferase, for all three mutants in *SI Appendix*, Fig. S5). The assays were done as time courses in reaction medium containing 10 mM MgCl_2_ in the absence or presence of 1 mM MnCl_2_, which enhances the DNA polymerase and strand annealing activities of WT G2L4 RT (21). In the absence of Mn^2+^, primer extension, snap-back DNA synthesis, MMEJ, and most terminal transferase activities were higher for the three RT3a mutants than those for the WT enzyme, indicating that mutations that destabilize RT3a interactions with the G2L4 RT active site make it easier for DNA substrates to displace the RT3a plug. Addition of Mn^2+^ increased the activity of the WT and mutant enzymes, but to a greater extent for the WT enzyme bringing its activity closer to that of the mutants, indicating that Mn^2+^ weakens the interaction of RT3a with the active site (Fig. 2*D* and *SI Appendix*, Fig. S5). Collectively, these findings indicate that the RT3a plug is an evolutionary adaptation that stabilizes the G2L4 RT apoenzyme in an inactive configuration that impedes promiscuous interactions with cellular nucleic acids until it encounters a physiological substrate.

### Cocrystal Structure of G2L4 RT Bound to a Snap-Back DNA Substrate.

Although several cryo-EM structures of group II intron RTs with bound group II intron RNAs have been determined (31–36), it has been difficult to obtain structures of group II intron RTs in an active conformation with bound template-primer and incoming dNTP, with GII RT being the only one for which such a structure has been determined (4). As an alternate approach for obtaining a structure of G2L4 RT in an active conformation, we returned to the previous finding that G2L4 RT has a strong strand-annealing activity that enables it to perform “snapback DNA synthesis,” a reaction in which the 3’ end of a DNA substrate folds back and searches for a short stretch of complementary upstream nucleotides for initiation of DNA synthesis (21).

**Fig. 3.**
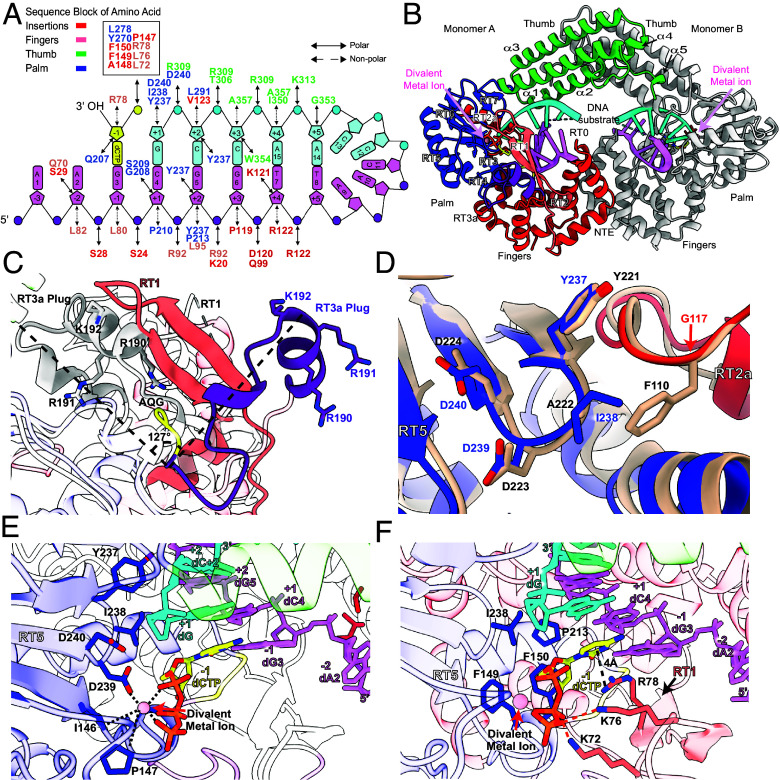
Structure of G2L4 RT in active conformation bound to a snapback DNA substrate and incoming dNTP. (*A*) Schematic of the snapback DNA substrate depicting interactions with G2L4 RT amino acid residues with names color-coded by their location within the RT as indicated at the *Top Left*. Bases, ribose rings, and phosphates numbered as indicated in the figure are represented by rectangles, pentagons, and circles, respectively, colored violet for the template strand, cyan for primer strand (including nucleotides incorporated from the crystallization mix), and yellow for the incoming dNTP. Double-arrowhead black lines and dashed line indicate polar and nonpolar interactions, respectively. (*B*) Structure of G2L4 RT snapback DNA synthesis complex, with monomer A regions colored as in Fig. 1*A*, monomer B colored gray, and divalent metal ions shown as pink spheres, most consistent with Mn^2+^ from the mother liquor according to CheckMyMetal Database (39). (*C*) Superimposition of G2L4 RT snapback complex and apoenzyme structures showing differences in RT1 (salmon) and the RT3a plug (purple) with the dashed black lines depicting the shift in position of the RT3a plug. Positively charged side chains in the extruded RT3a plug are shown as sticks with nitrogens (blue). (*D*) Superimposition of the active site of G2L4 RT in the snapback complex (palm, blue; RT2a, red) and GII RT (peach) with side chains of active-site residues shown as sticks colored by region with oxygens red. (*E*) Active-site interactions of G2L4 RT with a divalent metal ion (pink sphere) dipyramidally coordinated (dashed black lines) with active site residues and incoming dCTP shown as sticks colored by region as in panel *A* and dCTP with carbons, yellow; nitrogens, blue; phosphorous, orange, and oxygens, red. colored as panel *C* and *F*. Nucleotides in the snapback DNA are numbered and color-coded by location as in *A*. (*F*) Interactions between the incoming dCTP and RT1 are shown. Charged residues K72, K76, R78 from RT1 (salmon) and the hydrophobic residue F150 are displayed as sticks, while other residues are colored as in panel *E*. Salt bridge interactions are marked with orange dashed lines, and a potential base-stacking interaction between R78 and the incoming dCTP is indicated by a black dashed line.

Taking advantage of this activity, we obtained diffracting crystal of G2L4 RT bound to a 15-nt snapback DNA substrate (5’-AAGCGGTTAACCCAA-3’) that enabled initiation of DNA synthesis by base pairing of its two 3’ A residues (A14 and A15) to TT residues at upstream positions 7 and 8 (T7 and T8; Fig. 3*A*). To stabilize these short base-pairing interactions, the cocrystallization setup included Mn^2+^, which favors strand annealing by G2L4 RT (21), plus dCTP, and dideoxy GTP (ddGTP) to enable addition of three complementary nucleotides at positions +1 to +3 ending with ddGTP and followed by base pairing of an incoming dCTP opposite the templating base (G3 at position −1 in the snapback substrate). The crystal had a rod morphology and diffracted to 2.77 Å.

To solve the structure, we defined the fingers/palm and thumb as separate rigid bodies during molecular replacement to allow mobility between these two regions. This led to a solution in space group P2_1_2_1_2_1_, revealing a G2L4 RT dimer with each monomer bound to a separate 15-nt snapback DNA substrate per asymmetric unit (Fig. 3*B* and *SI Appendix*, Fig. S6 *A* and *B* and Table S1). The final model corresponds to full-length G2L4 RT protein with the DNA ligands fitted into the electron density corresponding to snapback DNAs. The two monomers were again virtually identical (RMSD = 0.78 Å).

As expected, the structure showed that A14 and A15 at the 3’ end of the snapback DNA substrate were annealed to upstream residues T8 and T7 (Fig. 3 *A* and *B*). The intervening AACCC loop (nucleotides 9 to 13) was not visible in the structure and presumably disordered. Because dCTP and ddGTP were included in the crystallization solution, two C residues complementary to G5 and G6 and ddGTP complementary to C4 of the template were added to the 3’ end of the DNA substrate, stabilizing the annealed microhomology (Fig. 3 *A* and *B*). dCTP complementary to G3 was bound at the active site but not incorporated due to lack of free hydroxyl groups on the preceding 3’-terminal dideoxyguanosine (Fig. 3*A*).

Compared to its occluded apoenzyme structure, each G2L4 RT monomer adopted an active configuration with the NTE moving outward to accommodate the bound DNA and the bound DNA substrate pushing the RT3a plug completely out of the active site (Fig. 3*C* and *SI Appendix*, Fig. S6*C*). Extrusion of the RT3a plug allowed the disordered RT1 in the apoenzyme structure to form the expected antiparallel β-strands over the active-site pocket as in other RTs and the AQG motif to revert to its functional position to coordinate alignment of the incoming dNTP, resulting in an active structure similar to that of GII RT (*SI Appendix*, Fig. S7*A*) (4). Except for a short two-turn α-helix (residues 186 to 193), the extruded RT3a plug became almost completely random coil, indicating that its interaction with the active site stabilized its more complex secondary and tertiary structures in the apoenzyme (Fig. 3*C*). Notably, three conserved basic residues of the RT3a plug (R190, R191, and K192), which interacted with the active site in the apoenzyme structure, were now exposed to bulk solvent, suggesting they might serve as initial contacts for sampling nucleic acid substrates (Fig. 3*C*).

### Configuration of the Active Site with Bound DNA Substrate and Incoming dNTP.

Although the active structures of G2L4 and GII RTs are similar (Fig. 3*D*), the snapback DNA synthesis structure revealed a series of structural adaptations that could optimize the DSBR activity of G2L4 RT. These include the conserved YIDD instead of YADD at the active site, a distinguishing feature of G2L4 RTs, with I favoring strand annealing in G2L4 RT and disfavoring primer extension in both G2L4 and GII RT (21). Except for the I/A substitution with compensations and consequences discussed further below, the configuration of the YIDD motif and its interactions with substrates closely resembled those of the YADD motif in GII RT (Fig. 3*D*; side-by-side comparisons in *SI Appendix*, Fig. S7 *B–E*) (4). The Y237 side chain hydroxyl group hydrogen bonds with the nucleotide base of the primer strand G5, and the first D (D239) of the YIDD motif coordinates a divalent metal ion dipyramidically with the phosphate group of the incoming dCTP (Fig. 3*E*). D240 faces outward away from the active site as seen in previous structures of GII RT and HIV RT (4, 26, 40).

The reformed RT1 lid over the active site stabilizes the triphosphate of the incoming nucleotide by salt bridges with positively charged residues K72, K76 (Fig. 3*F*). Notably, the side chain of R78 aligns parallel with the terminal base of the substrate (ddG+1) via a cation-π interaction of 4 Å, close to the base-pair distance of 3.4 Å (Fig. 3*F* and *SI Appendix*, Fig. S7*D*). This favorable interaction likely stabilizes the binding of the base of the incoming dNTP and may act as a bookend to push it forward into the active site aligned with the preceding base pairs. R78 in G2L4 RT corresponds to a conserved arginine residue that adopts the same configuration and likely plays a similar role to that of R75 in GII RT (*SI Appendix*, Figs. S1*C* and S7*E*) as well as HIV-1 RT (R72), and Hepatitis C Virus (HCV) RdRP (R158) (4, 26, 41). Further stabilization of the incoming dNTP in G2L4 RT comes from F150 of RT3, which interacts hydrophobically with the ribose sugar pucker of the incoming dCTP (Fig. 3*F* and *SI Appendix*, Fig. S7*D*). This phenylalanine, which is conserved in group II intron RTs (F143 in GII RT; *SI Appendix*, Figs. S1*C* and S7*E*) (4) and replaced by a tyrosine in HIV-1 RT (26), may play a role in stabilizing the incoming nucleotide during polymerization.

In addition to favoring strand annealing (21), the conserved active site I residue (I238) in the G2L4 RT YIDD motif potentially stabilizes the active configuration of the enzyme by fitting into a hydrophobic pocket formed by F149/150 and P213, and these residues engage in stacking hydrophobically with the sugar pucker of the base of the incoming dCTP (Fig. 3*F*), a stronger hydrophobic interaction than that for the conserved A residue in the YADD motif of GII RT (*SI Appendix*, Fig. S7*E*). The previous finding that I for A substitution in GII RT decreased the rate of primer extension (21) is likely due to van der Waals clashes of the substituted I residue with F110, which extends into the GII RT active site from RT2a (Fig. 3*D*) (4). In G2L4 RT, however, this phenylalanine in RT2a is replaced by a conserved glycine (G117), a covariation that leaves enough space for the isoleucine side chain to fit into the active site without steric hindrance (Fig. 3*D* and *SI Appendix*, Fig. S1*C*). The substitutions at the active site that enhance the DNA repair function of G2L4 RT may come at the expense of lower fidelity, a trade-off of human DNA polymerase θ and other DNA repair polymerases (42, 43).

### RT0-Loop and Thumb Domain Interactions That Favor Annealing of Microhomologies.

**Fig. 4.**
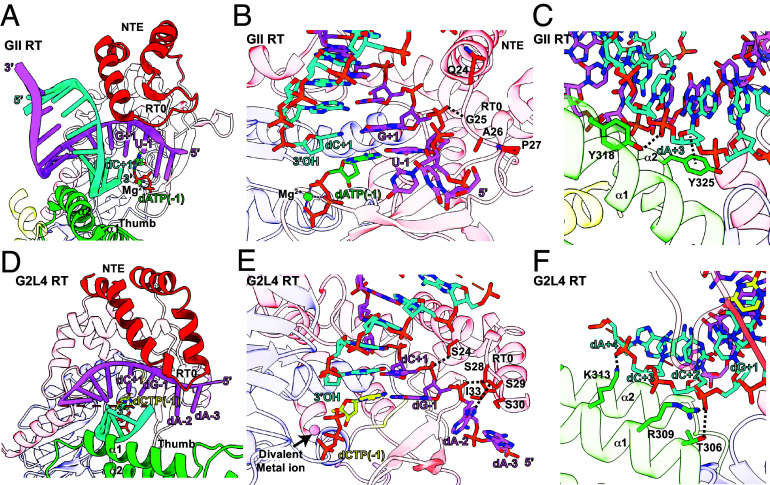
RT0-loop and thumb domain interactions with the annealed microhomology in the snapback DNA complex structure. (*A–C*) Wide-angle and close-up views of NTE/RT0 and thumb domain interactions for GII RT bound to annealed RNA template and DNA primer strands (violet and blue, respectively) and incoming dATP (stick, carbons, yellow; nitrogens, blue; phosphorus, orange; oxygens, red) (4). Numbers indicate nucleotide positions relative to the 5’ end of the template, with −1 corresponding to the position of the templating nucleotide and incoming dATP. (*D–F*) Wide-angle and close-up views of the NTE/RT0 and thumb domain interactions for G2L4 RT bound to a snapback DNA substrate with regions corresponding to the template strand (violet), primer strand (cyan), and incoming dCTP colored as Fig. 3*E*. Nucleotides and the incoming dNTP are numbered as in panel *A*.

The RT0 loop at the end of the NTE is a key structural feature of non-LTR-retroelement RTs that is required for the MMEJ activity but not the primer extension activity of both G2L4 and GII RTs (21). In the crystal structure of GII RT with a bound RNA template-DNA primer, the NTE inserts into the major groove of the template-primer duplex with the RT0 loop forming an 8-aa lid over the active site between positions −1 to +1, constraining the annealed template-primer and incoming dNTP (Fig. 4 *A* and *B*) (4). Additionally, two tyrosine residues in thumb helix α2 (Y318 and Y325) stabilize alignment of the DNA primer with the RNA template via phosphate backbone hydrogen bond and π-π stacking interactions, respectively (Fig. 4 *A* and *C*) (4).

For comparisons with GII RT, we focused on the corresponding regions of the G2L4 RT snapback DNA structure in which the 3’ end of the DNA served as a primer strand that initiated DNA synthesis by annealing to a short (2 nt) microhomology within an upstream region of the DNA substrate that served as the template strand. These comparisons showed that the longer NTE of G2L4 RT extended farther out over the single-stranded region of template strand to position −3 with its smaller RT0 loop (5 aa in both the snapback and apoenzyme structures) and flanking regions binding directly to the phosphate backbone between positions +1 and −2 (Fig. 4 *D* and *E*). The alignment of the DNA primer strand at the annealed microhomology differed from GII RT in being stabilized by hydrogen bond and salt bridge interactions with T306, R309, and K313 at the bottom of thumb helix α1 (Fig. 4 *D* and *F*).

A further adaptation of the NTE/RT0 loop for the DSBR function of G2L4 RT was a set of four serine residues (S24 in the NTE and S28-S30 at the center of the RT0 loop) that are strongly conserved in G2L4 RTs, but not in GII or other group II intron or non-LTR-retroelement RTs (*SI Appendix*, Figs. S1*C* and S8) (21). The snapback DNA synthesis structure indicated that hydroxyl side chains of these serine residues play a major role in DSBR by binding directly to the extended single-strand region of the template strand (Fig. 4 *D* and *E*). S28 hydrogen bonds to the backbone phosphate between dG−1 and dA−2, while S29 hydrogen bonds to the dA−2 base (Fig. 4*E*). S30, while not directly interacting with the template strand, forms an intramolecular hydrogen bond with the backbone nitrogen of I33 to stabilize a β-turn that protrudes S28 and S29 to interact with the template strand (Fig. 4*E*). S24 in the NTE forms a hydrogen bond with the phosphate backbone between dG−1 and dC+1, further stabilizing the extended interaction between the NTE/RT0 loop and the template strand (Fig. 4*E*).

To assess the functional importance of S28-S30 in the RT0 loop, we constructed a G2L4 RT mutant in which all three residues were changed to glycine residues (S3/G3). Biochemical assays (*SI Appendix*, Fig. S9 *A* and *B*) showed that this substitution for three serine residues inhibited most biochemical activities of G2L4 RT: primer extension (~threefold in the absence of Mn^2+^ and 100-fold in the presence of Mn^2+^); snapback DNA synthesis (sixfold in the absence of Mn^2+^ and threefold in the presence of Mn^2+^); MMEJ (roughly equal in the absence of Mn^2+^ and fourfold lower in the presence of Mn^2+^), with similar results for terminal transferase with different dNTPs. These findings suggest that mutations in the conserved RT0 loop serine residues can affect diverse biochemical activities, either directly or indirectly by mispositioning the NTE/RT0 loop. Collectively, the structural and biochemical findings indicate that the NTE/RT0 loop of G2L4 RTs evolved to play a critical role in MMEJ that differs mechanistically from that of GII RT.

### Mutations That Weaken Dimerization Affect Enzymatic Activities of G2L4 RT.

**Fig. 5.**
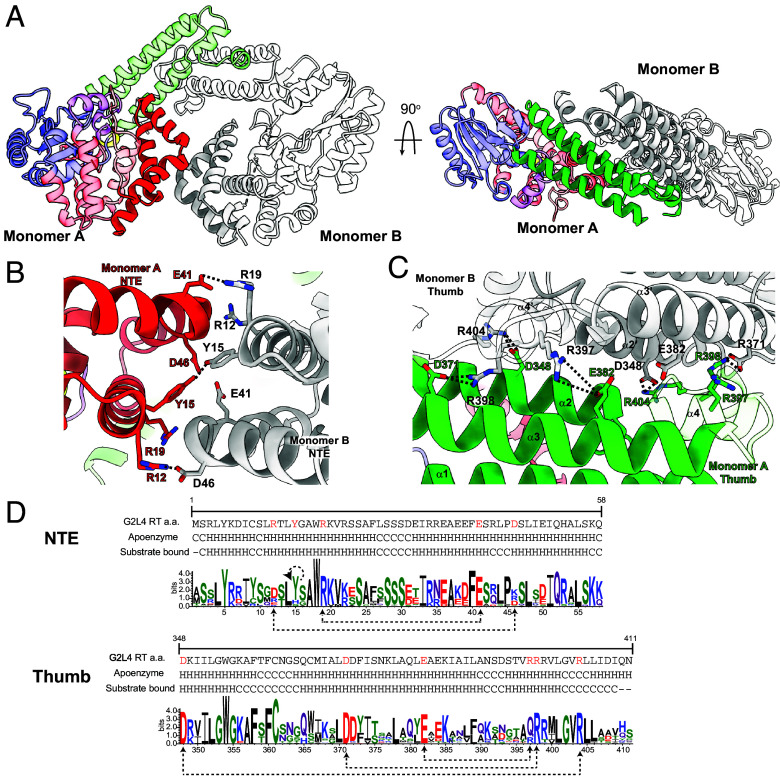
Structural and mutational analysis of interactions at the G2L4 RT dimer interface. (*A*) G2L4 RT dimer showing interacting regions of monomer A and B NTEs (*Left*) and a 90°-rotated view (*Right*) showing interacting regions of monomer A and B thumb domains. Regions in the left-hand monomer are colored as in Fig. 1*A*. (*B*) Interactions of NTE residues R12, Y15, and R19 of monomer A (red) with NTE residues D46, Y15, and E41 of monomer B (white). Black dashed lines indicate salt bridges or hydrogen bonds. (*C*) Thumb domain residues R397, R398, and R404 from monomers A (green) and B (gray) interact with E382, D371, and D348 from the opposite monomers, respectively. Black dashed lines indicate salt bridges or hydrogen bonds. Interacting residues are shown as sticks with carbons colored by monomer region with nitrogens and oxygens colored blue and red, respectively. (*D*) WebLogos depicting conservation of amino acids in interacting regions of the NTEs and the thumb domains of G2L4 RT dimers in the apoenzyme and snap-back DNA complex structures. H and C indicate amino acids in α-helical and random coil regions, respectively. Interactions between amino acid residues in the two monomers are indicated by red letters and black dashed lines with a black dashed circular line above Y15 indicating interactions between the same residue in the two monomers.

The G2L4 dimer interface in both the apoenzyme and cocrystal structures involve similar interactions (ionic, hydrogen bond, and hydrophobic) between the outer surfaces of the NTE and thumb of the two monomers, with the thumb domains contributing the larger area to the dimer interface (Fig. 5 *A*–*C*). This larger thumb domain interface reflects in part that the α-helical bundle comprising the thumb of G2L4 RT extends straight up 15 Å farther than in group IIC intron RTs in which a shorter α-helical bundle interacts with the DNA-binding domain to form a basic cleft that binds to a DNA hairpin structure at mobile group II intron-insertion sites (Fig. 1 *C* and *D*) (4, 34, 44). The G2L4 RT thumb also has a 22-aa C-terminal extension (residues 390 to 411) that includes two short α-helices (α4 residues 394 to 401 and α5 residues 407 to 411) that interact with the thumb domain of the opposite monomer (Fig. 1 *B* and *C*). We confirmed that G2L4 RT remains a dimer in the absence of the N-terminal MBP tag, although removal of the tag decreased the thermostability of the protein measured by DSF, reflecting a stabilizing effect of the MBP tag on its overall structure (*SI Appendix*, Fig. S10 *A* and *B*). Deletion of C-terminal residues 401 to 411, which include α5, likewise decreased protein stability, but did not prevent dimer formation.

To test whether dimerization of G2L4 RT is functionally important, we constructed three G2L4 RT mutants in which NTE and/or thumb residues identified as contributing to interactions that potentially stabilize the dimer interface were replaced by alanine residues: i) NTE residues R12, Y15, and R19 (denoted NTE); ii) Thumb residues R397 and R398 in α4 and R404 in a random coil region between α4 and α5 (denoted Thumb); and iii) NTE + Thumb residues R12, Y15, and R398 (denoted NTE+T; Fig. 5 *B*–*D*). Analysis of the mutant proteins on a nondenaturing gel showed that significant proportions of the NTE, Thumb, and NTE+T mutant proteins ran as monomers (12 ± 6%, 35 ± 8% and 54 ± 7%, respectively), while the WT and other G2L4 RT mutants analyzed in this study, as well as G2L4 RT with an A instead of an I at the active site, ran predominantly as dimers (≤7 ± 3% monomers; *SI Appendix*, Fig. S10*C*). All three of the dimer interface mutants had decreased thermostability, with the NTE + T and Thumb mutants having lower melting temperature than the NTE mutant (*SI Appendix*, Fig. S10*D*). These findings indicate that dimerization contributes to the stability of G2L4 apoenzymes and that the tested thumb domain interactions involving α4 and the random coil region make a stronger contribution to dimerization and protein stability than the tested NTE interactions.

Biochemical assays showed that the three dimer interface mutants had lower primer extension, snap-back DNA synthesis, MMEJ, and terminal transferase activities than did the WT enzyme in the absence or presence of Mn^2+^, but surprisingly, these decreases were more pronounced for the NTE and NTE+T mutants than for the Thumb domain mutant in most assays (*SI Appendix*, Fig. S11 *A* and *B*). While all three dimer interface mutants had substantial primer extension activity and close to WT snap-back DNA synthesis activity in the presence of Mn^2+^, the NTE and NTE+T mutants had little if any MMEJ activity in the presence or absence of Mn^2+^. Collectively, these findings indicate that mutations of interacting residues at the dimer interface affect the overall stability and biochemical activities of G2L4 RT, but with the NTE mutations, which have less effect on dimerization, having a larger effect on MMEJ than other biochemical activities, a finding relevant to the model for the DSBR mechanism described below.

### Model for MMEJ by G2L4 RT.

**Fig. 6.**
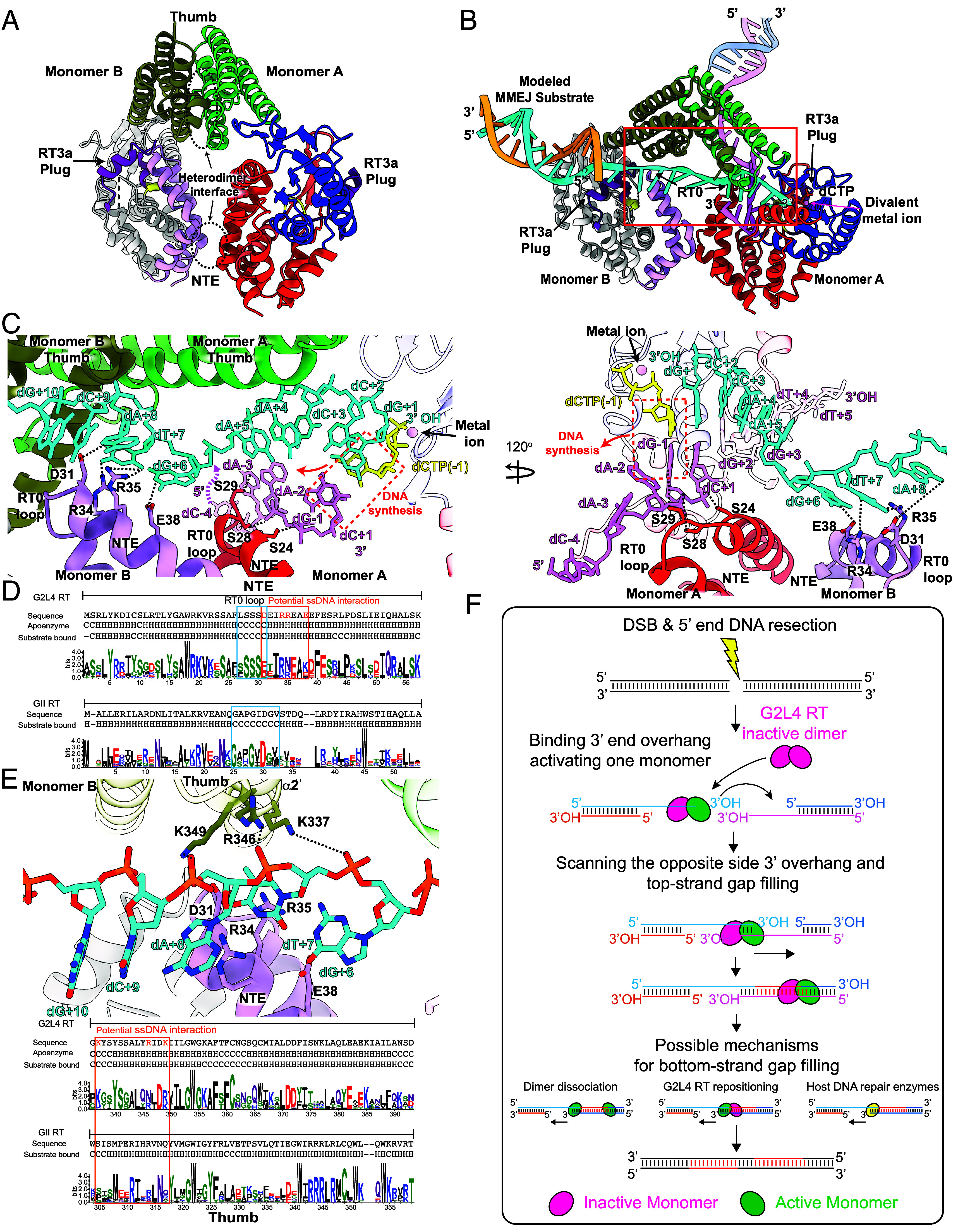
Model of the G2L4 RT MMEJ mechanism. (*A*) Model of a G2L4 RT heterodimer with active monomers A and inactive monomer B based on the G2L4 RT snapback structure and apoenzyme structure, respectively. The heterodimer interfaces between NTE and thumb regions are encircled by black dashed lines. Regions of monomer A are colored as in Fig. 1*A*, and the NTE/RT0 and thumb of monomer B are colored light purple and olive, respectively. The RT3a plug is colored purple in both monomers. (*B*) Model of a G2L4 RT dimer composed of a leading activated monomer A (*Right*) and a trailing inactive monomer B (*Left*) bound to the 3’ end of a single-stranded 3’-DNA overhang on the left side of a DSB (cyan) annealed to a 5-bp microhomology at the 3’ end of the 3’-overhang on the right side of the DSB (violet). Regions of monomer A are colored as in Fig. 1*A*, and the NTE/RT0 and thumb of monomer B are colored light purple and olive, respectively. RT3a plug, purple in both monomers. Regions of G2L4 RT interacting with the MMEJ substrate are highlighted in a red box. (*C*) *Left*, close-up view of panel *B* highlighting interactions (dashed black lines) of the leading active monomer and trailing inactive monomer with different regions of the MMEJ substrate. Conserved serine residues in the NTE/RT0 loop of the leading active monomer interact with dA−2 to dC+1 of the 3’-overhang on the right side of the DSB (violet). The positioning of the inactive trailing monomer shows potential contacts with the left-side 3’-overhang (cyan) without further adjustment. Base pairing of incoming dCTP (stick, yellow) with dG−1 of the template strand (violet) is highlighted in an orange box, and a red arrow indicates the direction of DNA synthesis. Upstream regions beyond dC−4 (dashed violet line) are omitted for clarity. *Right*, close-up of 120° horizontally rotated views highlighting interactions (dashed black lines) of the leading active monomer and trailing inactive monomer with different regions of the MMEJ substrate. (*D*) WebLogos comparing conservation of amino acids in the NTE/RT0 loop regions of G2L4 RTs to those of GII RTs. Amino acid residues that potentially interact with the single-stranded DNA are highlighted in orange. (*E*) *Top*, model of interactions between amino acid residues in the thumb domain of the trailing inactive monomer and the single-stranded gap between the active and inactive monomers in 3’-overhang on the left side of a DSB, colored as panel *B*. *Bottom*, WebLogos comparing conservation of thumb domain residues in G2L4 RTs to those in GII RTs, with those in G2L4 RT that potentially interact with nucleotide residues in the single-stranded gap highlighted in orange. H and C indicate amino acid residues in α-helical and random coil regions of the indicated structures. (*F*) Schematic of the hypothesized mechanism by which G2L4 RT functions in DSBR by MMEJ.

The snapback DNA synthesis structure revealed a mechanism relevant to MMEJ in which a G2L4 RT dimer bound the 3’ end of a single-stranded DNA ending with 3’ AA residues (primer strand) and then searched upstream regions (template strand) for complementary TT residues to which it could base pair for initiation of DNA synthesis (Fig. 3). At the high substrate concentrations used for crystallization, both monomer subunits of the same dimer had activated structures bound to separate short snapback DNA substrates, but we were unable to obtain crystal with longer snapback substrates under the same or different conditions. Despite conformational changes during substrate binding and initiation of DNA synthesis coupled to the reconfiguration of RT3a, there were no significant differences in the dimer interfaces between apoenzyme or substrate-bound G2L4 RT monomers (Fig. 6*A*). This finding suggested that G2L4 RT could remain a stable dimer with one monomer active and the other remaining inactive.

Under physiological conditions where DNA substrate binding is likely rate limiting, activation of both monomers by binding to the 3’ end of a single-strand DNA overhang at a DSB site likely occurs sequentially if at all. Utilizing the snapback complex and apoenzyme structures, we modeled a G2L4 RT dimer bound to the 3’ end of the 3’-overhang on the left side of a DSB (primer strand, cyan) annealed to a 5-bp microhomology at the 3’ end of the 3’-overhang on the right side of the DSB (template strand, violet; Fig. 6 *B* and *C*). In this model, the leading right-hand monomer (monomer A, regions colored as in Fig. 1*A*) is activated by binding the 3’ end of the primer strand (cyan), while the trailing left-hand monomer (monomer B, gray with NTE/RT0 loop magenta and thumb α2 olive) remains inactive (Fig. 6*B*). The model, based entirely on the two structures without further adjustments, showed that the NTE/RT0 loops of both monomers were positioned by their interactions at the dimer interface on opposite sides of the annealed 5-bp microhomology, with the leading active monomer poised to fill in the 3’-overhang on the right side of the DSB (Fig. 6*B*). Notably, the extruded RT3a plug of the active monomer is located in a forward position near the independently modeled single-stranded 3’-overhang on the opposite side of the DSB, consistent with a potential contribution to MMEJ (Fig. 6*B*).

In a close-up view, the NTE/RT0 loop of the leading active monomer bound to the 3’-overhang on the left side of the DSB (primer strand, cyan) is positioned over the annealed microhomology in the 3’-overhang on the right side (template strand, violet) (Fig. 6*C*). This positioning is stabilized by phosphate backbone interactions between conserved serine residues and nucleotides dA−2 to dC+1, as seen in the snapback DNA complex structure (Fig. 4 *E* and *F*). With this position fixed, the NTE/RT0 of the trailing inactive monomer was positioned to interact with three exposed bases (dG+6 to dA+8) of the priming strand (cyan) in the gap between the two monomers via hydrogen bonds from D31, R34, R35, and E38 (Fig. 6*C*). Supporting these interactions: i) charged residues at these positions in the NTE were conserved in G2L4 RTs, while only the cognate of D31 (D30 in GII RT) was conserved in the larger RT0 loop of GII RTs (Fig. 6*D*), and ii) mutations in which R35 by itself or together with R34 or E38 were replaced by alanine residues preferentially inhibited MMEJ (*SI Appendix*, Fig. S12*A*). Combined R34A/D31A mutations had little or no effect on MMEJ or primer extension in vitro, suggesting a smaller contribution of these residues (*SI Appendix*, Fig. S12*A*). A critical role for optimal positioning of the NTE/RT0 loops of both monomers on an MMEJ substrate was further supported by the finding above that mutations that affect NTE interactions at the dimer interface almost totally abolished MMEJ activity, while having less effect on other biochemical activities, including retaining relatively high primer extension and snapback DNA synthesis activity in the presence of Mn^2+^ (*SI Appendix*, Fig. S11*A*).

At an earlier stage of MMEJ, the binding of a G2L4 RT dimer may stabilize the single-stranded 3’-overhang on the left side of the DSB (priming strand) in an extended conformation that facilitates scanning for and annealing to a microhomology in the 3’-overhang on the opposite side of the DSB. In addition to the above interactions, the model with no further adjustments suggests that the α2-helix of the thumb domain of the inactive monomer (olive) may contribute to this function by phosphate–backbone interactions of K337, R346, and K349 with dG+6, dT+7, and dA+8 of the 3’-overhang from the left side of the DSB (Fig. 6*E*). These amino acid residues, which do not contribute to the dimer interface, were conserved as positively charged residues in G2L4 RTs but not GII RTs (Fig. 6 *E*, *Bottom*). A model with both monomers in an active conformation displaced the RT0 loop of the trailing monomer away for the MMEJ substrate (*SI Appendix*, Fig. S12*B*), and a model with a leading active and trailing inactive monomer showed a steric clash with annealed microhomology >6 bp (*SI Appendix*, Fig. S12*C*), consistent with G2L4 RT’s preference for shorter annealed microhomologies and longer single-strand gaps than GII RT, which functions as a monomer (21).

The model for the initial steps of MMEJ by G2L4 RT positions the enzyme to fill in the single-stranded gap on the right side of the DSB (Fig. 6*F*). This step is potentially critical for cell viability by generating a continuous single-stranded DNA across the annealed microhomology between the two 3’-overhangs at the DSB. The remaining single-stranded gap on the left side of the DSB could be filled in by several possible mechanisms, including: i) dissociation and activation of the trailing monomer; ii) repositioning of the first or recruitment of a second partially activated dimer; or iii) by cellular DNA repair enzymes, which are also needed to fully repair the DSB (Fig. 6*F*).

## Discussion

Here, we obtained crystal structures of a chromosomally encoded G2L4 RT apoenzyme and a G2L4 RT snapback DNA synthesis complex, revealing features that evolved to optimize its cellular function in DSBR via MMEJ and suggesting a model for the MMEJ mechanism (Fig. 6*F*). After synthesis, the G2L4 RT apoenzyme forms a homodimer with each subunit stabilized by the RT3a plug, a novel structural feature of G2L4 RTs not predicted by the most recent version of AlphaFold 3.0. The G2L4 RT apoenzyme structure showed that the RT3a plug completely blocks the active site of each monomer, displacing catalytic divalent metal ions and rendering the enzyme inactive until displaced by a physiological substrate (Figs. 1*E* and 2 *B* and *C*). Mutations that weakened the interaction of the RT3a plug with the RT active site led to higher biochemical activities across all tested substrates, suggesting an equilibrium between an inactive and an activatable conformation that can lead to full binding of a physiological substrate (Fig. 2*D* and *SI Appendix*, Fig. S5). In the active conformation, conserved basic residues of the RT3a plug that block the G2L4 RT active site move toward the surface of the protein (Fig. 3*C*), potentially enabling sampling of nucleic acid substrates by nonspecific binding.

The snapback DNA synthesis structure indicated a mechanism relevant to MMEJ in which a G2L4 RT dimer binds the 3’ end of a single-stranded DNA substrate that serves as a primer and searches for complementary microhomologies in upstream regions of the snapback substrate that serves as a template (Fig. 3). This structure provided direct evidence that G2L4 RT functions as dimer in a reaction akin to MMEJ, but with both monomers activated at high concentrations of short DNA substrate that could not reach the dimer interface, and we were unable to obtain crystals with longer DNA substrates that would extend across the dimer interface. Modeling suggested that the conserved dimer interface of G2L4 RT enables it to bind to a longer single-strand 3’-DNA overhang on one side of a DSB site as a heterodimer consisting of a leading monomer activated by binding the 3’ end of the 3’-overhang as a primer and a trailing monomer that remains inactive, likely reflecting the situation at lower substrate concentrations in vivo (Fig. 6*F*, step 1). In this configuration, the NTE/RT0 loop of both G2L4 RT monomers would contribute to stabilizing the 3’ overhang on one side of the DSB in an extended conformation with the leading active monomer positioned to search for and promote annealing to a microhomology in the 3’ overhang on the opposite side of the DSB (Fig. 6*F*, step 2).

Our structural and biochemical analyses revealed a series of conserved structural features that evolved to optimize the DSBR activity of G2L4 RT. These included the conserved I instead of A in the YxDD motif at the active site of G2L4 RT (21), which was found previously to favor strand annealing required for MMEJ over primer extension. The snapback DNA synthesis structure showed that I238 forms a hydrophobic stacking interaction with the sugar pucker of the G+1 base, playing a pivotal role in securing the substrate within the active site (Fig. 3 *E* and *F*). This interaction helps maintain the nucleotide’s proper orientation, which is essential for accurate catalysis. I instead of A at the active site also favors the use of short annealed primers (2 to 5 nt) and strongly disfavors the use of longer annealed primers ≥10 nt (21), consistent with the preference of the G2L4 RT MMEJ activity for shorter annealed microhomologies than GII RT (21). Despite remaining a stable dimer (*SI Appendix*, Fig. S10*C*), a G2L4 RT mutant with A instead of I at the active site enabled the use of longer primers and annealed microhomologies (21), likely due to disruption of the stabilizing active site interaction of the isoleucine side chain with the sugar pucker of the G+1 base (Fig. 3 *E* and *F*).

Other conserved structural features that optimize the DSBR activity of G2L4 RT included a longer NTE than that of GII RT with a smaller (5 nt) RT0 loop centered on three conserved serine residues found in the snapback DNA synthesis structure to bind and likely stabilize an annealed microhomology (Fig. 4*D*). This RT0 loop structure differs from that of GII RT, which forms a lid over the RT active site (positions −1 to +1; Fig. 4*A*), as well as those of human LINE-1 and insect R2 element RTs that have larger “lid over the RT active site” morphologies (*SI Appendix*, Fig. S13 *A*–*C*) (14, 15, 37). All three of these RTs as well as other retroelement RTs that associate with DSBs lack multiple conserved serine residues that contribute to the optimized DSBR activity of G2L4 RT (45–47). A recent study found that a retroviral RT could function in DSBR in mammalian cells by filling in short single-stranded DNA gaps, revealing a wider association of RTs with DNA repair (48).

Finally, the G2L4 RT structural features revealed in this study suggest ways of optimizing non-LTR-retroelement RTs for genome engineering applications. These include: i) modifications of RT3a that stabilize the free protein in an inactive configuration that prevents promiscuous reverse transcription of cellular RNAs until it encounters a physiological substrate; ii) an extended NTE with an RT0 loop containing residues that interact directly with a single-stranded DNA overhang to form a stabilizing handle that favors strand annealing; iii) the substitution of different residues at the x position Y/FxDD motif and its covarying partner in RT2a to modulate the balance between RT fidelity and strand annealing; and iv) modifications of the outer surfaces of the NTE and thumb to form a dimer interface, whose stability could be tuned to localize two non-LTR-retroelement RT monomers to function together or separately for genome engineering functions.

## Materials and Methods

Bacterial strains, DNA oligonucleotides, recombinant plasmids, protein purification, and structural, biochemical, and bioinformatic methods used in this study are described in *SI Appendix*.

## Supplementary Material

Appendix 01 (PDF)

## Data Availability

The X-ray crystal structures of G2L4 RT apoenzyme and active G2L4 RT bound to a snapback DNA substrate have been deposited in the Protein Data Bank (accession numbers PDB:9D5X (49) and PDB:9D4S (50), respectively). All raw images have been deposited at Mendeley Data, V3, DOI: 10.17632/r5hp4spdjx.3 (51). All other data are included in the manuscript and/or *SI Appendix*.

## References

[1] Y. Xiong, T. H. Eickbush, Origin and evolution of retroelements based upon their reverse transcriptase sequences. EMBO J. **9**, 3353–3362 (1990). 1698615 10.1002/j.1460-2075.1990.tb07536.x PMC552073

[2] H. Wang, A. M. Lambowitz, The Mauriceville plasmid reverse transcriptase can initiate cDNA synthesis de novo and may be related to reverse transcriptase and DNA polymerase progenitor. Cell **75**, 1071–1081 (1993). 7505202 10.1016/0092-8674(93)90317-j

[3] H. S. Malik, W. D. Burke, T. H. Eickbush, The age and evolution of non-LTR retrotransposable elements. Mol. Biol. Evol. **16**, 793–805 (1999). 10368957 10.1093/oxfordjournals.molbev.a026164

[4] J. L. Stamos, A. M. Lentzsch, A. M. Lambowitz, Structure of a thermostable group II intron reverse transcriptase with template-primer and its functional and evolutionary implications. Mol. Cell **68**, 926–939.e4 (2017). 29153391 10.1016/j.molcel.2017.10.024 PMC5728383

[5] K. K. Kojima, M. Kanehisa, Systematic survey for novel types of prokaryotic retroelements based on gene neighborhood and protein architecture. Mol. Biol. Evol. **25**, 1395–1404 (2008). 18391066 10.1093/molbev/msn081

[6] D. M. Simon, S. Zimmerly, A diversity of uncharacterized reverse transcriptases in bacteria. Nucleic Acids Res. **36**, 7219–7229 (2008). 19004871 10.1093/nar/gkn867 PMC2602772

[7] N. Toro, R. Nisa-Martínez, Comprehensive phylogenetic analysis of bacterial reverse transcriptases. PLoS One **9**, e114083 (2014). 25423096 10.1371/journal.pone.0114083 PMC4244168

[8] S. Zimmerly, L. Wu, An unexplored diversity of reverse transcriptases in bacteria. Microbiol. Spectr. **3**, 3.2.13 (2015). 10.1128/microbiolspec.MDNA3-0058-2014 26104699

[9] T. Cavalier-Smith, Intron phylogeny: A new hypothesis. Trends Genet. **7**, 145–148 (1991). 2068786

[10] R. J. Grainger, J. D. Beggs, Prp8 protein: At the heart of the spliceosome. RNA **11**, 533–557 (2005). 15840809 10.1261/rna.2220705 PMC1370742

[11] W. Martin, E. V. Koonin, Introns and the origin of nucleus-cytosol compartmentalization. Nature **440**, 41–45 (2006). 16511485 10.1038/nature04531

[12] W. P. Galej, C. Oubridge, A. J. Newman, K. Nagai, Crystal structure of Prp8 reveals active site cavity of the spliceosome. Nature **493**, 638–643 (2013). 23354046 10.1038/nature11843 PMC3672837

[13] A. M. Lambowitz, M. Belfort, Mobile bacterial group II introns at the crux of eukaryotic evolution. Microbiol. Spectr. **3**, 3.1.04 (2015). 10.1128/microbiolspec.MDNA3-0050-2014 PMC4394904 26104554

[14] A. Thawani, A. J. F. Ariza, E. Nogales, K. Collins, Template and target-site recognition by human LINE-1 in retrotransposition. Nature **626**, 186–193 (2024). 38096901 10.1038/s41586-023-06933-5 PMC10830416

[15] E. T. Baldwin , Structures, functions and adaptations of the human LINE-1 ORF2 protein. Nature **626**, 194–206 (2024). 38096902 10.1038/s41586-023-06947-z PMC10830420

[16] S. Doulatov , Tropism switching in Bordetella bacteriophage defines a family of diversity-generating retroelements. Nature **431**, 476–481 (2004). 15386016 10.1038/nature02833

[17] M.-C. Chopin, A. Chopin, E. Bidnenko, Phage abortive infection in lactococci: Variations on a theme. Curr. Opin. Microbiol. **8**, 473–479 (2005). 15979388 10.1016/j.mib.2005.06.006

[18] S. Silas , Direct CRISPR spacer acquisition from RNA by a natural reverse transcriptase–Cas1 fusion protein. Science **351**, aad4234 (2016). 26917774 10.1126/science.aad4234 PMC4898656

[19] A. Millman , Bacterial retrons function in anti-phage defense. Cell **183**, 1551–1561.e12 (2020). 33157039 10.1016/j.cell.2020.09.065

[20] L. Gao , Diverse enzymatic activities mediate antiviral immunity in prokaryotes. Science **369**, 1077–1084 (2020). 32855333 10.1126/science.aba0372 PMC7985843

[21] S. K. Park, G. Mohr, J. Yao, R. Russell, A. M. Lambowitz, Group II intron-like reverse transcriptases function in double-strand break repair. Cell **185**, 3671–3688.e23 (2022). 36113466 10.1016/j.cell.2022.08.014 PMC9530004

[22] G. Mohr, J. Yao, S. K. Park, L. Markham, A. M. Lambowitz, Mechanisms used for cDNA synthesis and site-specific integration of RNA into DNA genomes by a reverse transcriptase-Cas1 fusion protein. Sci. Adv. **10**, eadk8791 (2024). 38608016 10.1126/sciadv.adk8791 PMC11014452

[23] S. Tang , De novo gene synthesis by an antiviral reverse transcriptase. Science **386**, eadq0876 (2024). 39116258 10.1126/science.adq0876 PMC11758365

[24] M. E. Wilkinson, D. Li, A. Gao, R. K. Macrae, F. Zhang, Phage-triggered reverse transcription assembles a toxic repetitive gene from a noncoding RNA. Science **386**, eadq3977 (2024). 39208082 10.1126/science.adq3977 PMC12039810

[25] P. Argos, A sequence motif in many polymerases. Nucleic Acids Res. **16**, 9909–9916 (1988). 2461550 10.1093/nar/16.21.9909 PMC338826

[26] L. A. Kohlstaedt, J. Wang, J. M. Friedman, P. A. Rice, T. A. Steitz, Crystal structure at 3.5 A resolution of HIV-1 reverse transcriptase complexed with an inhibitor. Science **256**, 1783–1790 (1992). 1377403 10.1126/science.1377403

[27] E. Arnold , Structure of HIV-1 reverse transcriptase/DNA complex at 7 Å resolution showing active site locations. Nature **357**, 85–89 (1992). 1374166 10.1038/357085a0

[28] F. J. H. Blocker , Domain structure and three-dimensional model of a group II intron-encoded reverse transcriptase. RNA **11**, 14–28 (2005). 15574519 10.1261/rna.7181105 PMC1370687

[29] S.-Q. Gu , Genetic identification of potential RNA-binding regions in a group II intron-encoded reverse transcriptase. RNA **16**, 732–747 (2010). 20179150 10.1261/rna.2007310 PMC2844621

[30] S. Mohr , Thermostable group II intron reverse transcriptase fusion proteins and their use in cDNA synthesis and next-generation RNA sequencing. RNA **19**, 958–970 (2013). 23697550 10.1261/rna.039743.113 PMC3683930

[31] G. Qu , Structure of a group II intron in complex with its reverse transcriptase. Nat. Struct. Mol. Biol. **23**, 549–557 (2016). 27136327 10.1038/nsmb.3220 PMC4899178

[32] D. B. Haack , Cryo-EM structures of a group II intron reverse splicing into DNA. Cell **178**, 612–623.e12 (2019). 31348888 10.1016/j.cell.2019.06.035 PMC6662634

[33] N. Liu , Exon and protein positioning in a pre-catalytic group II intron RNP primed for splicing. Nucleic Acids Res. **48**, 11185–11198 (2020). 33021674 10.1093/nar/gkaa773 PMC7641739

[34] K. Chung , Structures of a mobile intron retroelement poised to attack its structured DNA target. Science **378**, 627–634 (2022). 36356138 10.1126/science.abq2844 PMC10190682

[35] L. Xu, T. Liu, K. Chung, A. M. Pyle, Structural insights into intron catalysis and dynamics during splicing. Nature **624**, 682–688 (2023). 37993708 10.1038/s41586-023-06746-6 PMC10733145

[36] D. B. Haack, B. Rudolfs, C. Zhang, D. Lyumkis, N. Toor, Structural basis of branching during RNA splicing. Nat. Struct. Mol. Biol. **31**, 179–189 (2024). 38057551 10.1038/s41594-023-01150-0 PMC10968580

[37] M. E. Wilkinson, C. J. Frangieh, R. K. Macrae, F. Zhang, Structure of the R2 non-LTR retrotransposon initiating target-primed reverse transcription. Science **380**, 301–308 (2023). 37023171 10.1126/science.adg7883 PMC10499050

[38] C. Zhao, A. M. Pyle, Crystal structures of a group II intron maturase reveal a missing link in spliceosome evolution. Nat. Struct. Mol. Biol. **23**, 558–565 (2016). 27136328 10.1038/nsmb.3224 PMC4899126

[39] H. Zheng , Validation of metal-binding sites in macromolecular structures with the CheckMyMetal web server. Nat. Protoc. **9**, 156–170 (2014). 24356774 10.1038/nprot.2013.172 PMC4410975

[40] K. Das, S. E. Martinez, R. P. Bandwar, E. Arnold, Structures of HIV-1 RT-RNA/DNA ternary complexes with dATP and nevirapine reveal conformational flexibility of RNA/DNA: Insights into requirements for RNase H cleavage. Nucleic Acids Res. **42**, 8125–8137 (2014). 24880687 10.1093/nar/gku487 PMC4081091

[41] T. C. Appleby , Viral replication. Structural basis for RNA replication by the hepatitis C virus polymerase. Science **347**, 771–775 (2015). 25678663 10.1126/science.1259210

[42] M. E. Arana, M. Seki, R. D. Wood, I. B. Rogozin, T. A. Kunkel, Low-fidelity DNA synthesis by human DNA polymerase theta. Nucleic Acids Res. **36**, 3847–3856 (2008). 18503084 10.1093/nar/gkn310 PMC2441791

[43] W. Yang, Y. Gao, Translesion and repair DNA polymerases: Diverse structure and mechanism. Annu. Rev. Biochem. **87**, 239–261 (2018). 29494238 10.1146/annurev-biochem-062917-012405 PMC6098713

[44] S. K. Park, “Structure-function analysis of group II intron and related bacterial reverse transcriptases,” PhD thesis, The University of Texas at Austin, Austin, TX (2022).

[45] S. C. Teng, B. Kim, A. Gabriel, Retrotransposon reverse-transcriptase-mediated repair of chromosomal breaks. Nature **383**, 641–644 (1996). 8857543 10.1038/383641a0

[46] J. K. Moore, J. E. Haber, Capture of retrotransposon DNA at the sites of chromosomal double-strand breaks. Nature **383**, 644–646 (1996). 8857544 10.1038/383644a0

[47] T. A. Morrish , Endonuclease-independent LINE-1 retrotransposition at mammalian telomeres. Nature **446**, 208–212 (2007). 17344853 10.1038/nature05560

[48] C. Zheng, G. Zhang, L. J. Dean, E. J. Sontheimer, W. Xue, The reverse transcriptase domain of prime editors contributes to DNA repair in mammalian cells. Nat. Biotechnol., 1–8 (2025). 39962280 10.1038/s41587-025-02568-1 PMC13394396

[49] M. Guo, J. L. Stamos, Y. J. Zhang, Structure of G2L4 RT Apoenzyme. *Protein* Data Bank. https://www.rcsb.org/structure/9D5X. Deposited 14 August 2024.

[50] M. Guo, Y. J. Zhang, Structure of G2L4 RT in complex with 15 nucleotide snapback substrate. *Protein* Data Bank. https://www.rcsb.org/structure/9D4S. Deposited 12 August 2024.

[51] S. K. Park, Structural basis for the evolution of a domesticated group II intron-like reverse transcriptase to function in host cell DNA repair. Mendeley Data. https://data.mendeley.com/datasets/r5hp4spdjx/3. Deposited 20 May 2025. 10.1073/pnas.2504208122 PMC12337344 40729381

