# Supplementary material for: Structural basis for the evolution of a domesticated group II intron–like reverse transcriptase to function in host cell DNA repair
Source: Proc Natl Acad Sci U S A. 2025 Jul 29;122(31):e2504208122. doi: 10.1073/pnas.2504208122 (PMC12337344; doi:10.1073/pnas.2504208122)
Supplement: Supplementary file 1 — Appendix 01 (PDF) [file pnas.2504208122.sapp.pdf]

PNAS

SI Appendix for

Structural basis for the evolution of a domesticated group II intron-like reverse transcriptase to function in host cell DNA repair

Seung Kuk Park[a,1,2], Mo Guo[a,1], Jennifer L. Stamos[a], Wantae Kim[a], Sidae Lee[a], Y. Jessie Zhang[a,3] and Alan M. Lambowitz[a,b,3]

[a]Department of Molecular Biosciences, University of Texas at Austin, Austin, TX 78712

[b]Department of Oncology, Dell Medical School, University of Texas at Austin, 78712

[3]To whom correspondence may be addressed. Email: jzhang@cm.utexas.edu or lambowitz@austin.utexas.edu.

[1]S.K.P. and M.G. contributed equally to this work.

[2]Present address: Stanford Cancer Institute, Stanford University School of Medicine, Stanford, CA 94305.

This PDF file includes:

Materials and Methods

Figures S1 to S13

Tables S1 to S3

SI References

Materials and Methods

Bacterial Strains. *E. coli* HMS174 (DE3) (F^- recA1 hsdR (r_{K12}^- m_{K12}^+) Rif ^{R}; Novagen) was used for cloning of recombinant plasmids, and *E. coli* Rosetta 2 (F^- ompT hsdS_B (r_B^- m_B^-) gal dcm pRARE2 Cap^R; Novagen) was used for expression and purification of recombinant WT and mutants G2L4 RT originated from *Pseudomonas aeruginosa* AZPAE12409 strain.

Oligonucleotides. HPLC-purified oligonucleotides purchased from Integrated DNA Technologies (IDT) are listed in Table S2. For biochemical experiments, oligonucleotides were 5'-labeled with [γ-^{32}P]-ATP (6,000 Ci/mmol; Revvity) by using T4 polynucleotide kinase (New England Biolabs) followed by clean-up with an Oligo Clean & Concentrator Kit (Zymo Research) according to the manufacturer's protocols. Quantitation of labeled oligonucleotides was performed by using a Qubit ssDNA assay kit (Thermo Fisher Scientific) according to the manufacturer's protocol.

Recombinant Plasmids. Recombinant plasmids are listed in Table S3. pMal-RT recombinant plasmids used to express wild-type and mutant G2L4 RTs in *E. coli* were derivatives of pMal-c5X (New England Biolabs), which includes a factor Xa cleavable maltose-binding protein (MBP) tag, an Amp^R marker, and an isopropyl β-D-1-thiogalacto-pyranoside (IPTG)-inducible tac promoter (1). Plasmids expressing additional G2L4 RT mutant proteins in this study were constructed by using a Q5 mutagenesis kit (New England Biolabs).

Bioinformatics and Amino Acids Sequence Alignments for G2L4 and Other RTs. The amino acid sequences of GsI-IIC (GII) RT and G2L4 RTs were obtained from refs 4 and 20, respectively. WebLogos (2) were created for 130 different bacterial G2L4 RTs and 500 different bacterial GsI-IIC (GII) RTs that were identified by BLASTP (3) as having ≥50% sequence identity and then aligned using ClustalW through Jalview (4, 5). The NCBI accession numbers for other RTs were sourced from Zimmerly and Wu (2015) (6). Amino acids sequences of other RTs were obtained from NCIB or RCSB PDB (https://www.rcsb.org). Multiple sequence alignments of RTs were performed using T-Coffee through Jalview (4, 7).

Protein Purification. WT and mutant N-terminal MBP-tagged G2L4 RTs were purified as described (1). pMal-c5x Amp^R WT and mutant G2L4 RT expression plasmids were transformed into *E. coli* Rosetta 2 Cap^R cells (Novagen). The transformants were plated on LB agar plates containing carbenicillin (100 µg/mL) and chloramphenicol (25 µg/mL) and incubated overnight at 37°C. The next day, a single colony was picked, inoculated into 20 mL of LB media containing carbenicillin (100 µg/mL) and chloramphenicol (25 µg/mL), and incubated with shaking at 220 rpm for 14-16 h at 37°C. The 20 mL-overnight grown cells were transferred into 1 L of LB media containing carbenicillin (100 µg/mL) and chloramphenicol (25 µg/mL) and incubated with shaking at 220 rpm at 37°C until OD_{600} reached 0.8-1.0. Protein expression was then induced by adding 100 µM IPTG and incubating at 100 rpm for 19-21 h at 18°C. After centrifugation in a 50-mL conical tube (Sarstedt) at 4,000 x g for 25 min in an Avanti J-E centrifuge (Beckman Coulter), cell pellets were transferred into a second 50-mL conical tube and resuspended in 45 mL of lysis buffer containing 20 mM Tris-HCl pH 7.5, 500 mM NaCl, 0.1% Triton X-100, 0.1% ß-mercaptoethanol, and 20% glycerol. The resuspended cells were then sonicated three times for 1 min at 80% amplitude using a Branson Sonifier 250 (Branson Ultrasonics), followed by centrifugation at 15,500 x g for 25 min. After collecting the supernatant, 0.04% polyethyleneimine (final concentration) was added, and the tube was inverted two or three times and then placed on ice for 10 min to precipitate nucleic acids. Precipitates were removed by centrifugation at 15,500 x g for 10 min as above, and the supernatant was filtered through a 0.45-mm pore size PES membrane (Thermo Fisher Scientific). The filtered supernatants were

then loaded onto a 5-mL HiTrap MBP HP column (Cytiva) at flow rate of 5 mL/min using a AKTA™ start FPLC (Cytiva), and the column was washed sequentially with five column volumes of buffer A (20 mM Tris-HCl pH 7.5, 100 mM NaCl, 0.1% ß-mercaptoethanol, 10% glycerol) followed sequentially by five column volumes of washing buffer (20 mM Tris-HCl pH 7.5, 1.5 M NaCl, 0.1% Triton X-100, 0.1% ß- mercaptoethanol, and 10% glycerol) and five column volumes of buffer A. Column-bound protein was eluted with 10 column volumes of elution buffer (20 mM Tris-HCl pH 7.5, 100 mM NaCl, 0.1% ß-mercaptoethanol, 10% glycerol, and 10 mM maltose). Column fractions containing the RT protein were identified by analyzing 15-µL samples by SDS-PAGE and staining with 0.25% Coomassie brilliant blue R (Sigma-Aldrich). Pooled fractions containing the protein were then loaded onto a 5-mL HiTrap Heparin HP column (Cytiva) at flow rate of 5 mL/min using a AKTA™ start FPLC (Cytiva). After washing the column with five column volumes of buffer A, bound proteins were eluted using ten column volumes of an NaCl gradient between buffer A, which contains 100 mM NaCl, and buffer B, which contains 1.5 M NaCl (see above). Column fractions containing the RT protein were again sampled by SDS-PAGE as above and pooled fractions were concentrated using an Amicon® Ultra-15 (30k) concentrator (Millipore). The concentrated samples were then diluted in 20 mM Tris-HCl pH 7.5, 50 mM NaCl and 50% glycerol to a final concentration of 8-10 mg/ml and stored at -80℃ until used.

SDS- and Native-Polyacrylamide Gel Electrophoresis. For SDS-PAGE, WT or mutant G2L4 RTs (10 µg) were diluted in double-distilled H_2O up to 15 µL followed by adding 5 µL of 4X sample buffer (200 mM Tris-HCl pH 6.8, 400 mM dithiothreitol (DTT), 8% SDS, 6 mM bromophenol blue, 40% glycerol) and incubating at 95°C for 5 min. The samples were then loaded onto a NuPAGE 4-12% Bis-Tris gel with a 250 kDa Plus Prestained Protein Marker (Vazyme) in a parallel lane and run in 1X MES running buffer (Thermo Fisher Scientific) at 150 V for 1 h by using an XCell Surelock Electrophoresis Cell according to the manufacturer's protocol. For native gels, WT or mutant G2L4 RTs (1 µg/14 µL) were mixed with 3 µL of 6X native gel sample buffer (300 mM Tris-HCl pH 8.5, 60% glycerol) and 1 µL of 1% Coomassie Brilliant Blue G-250 solution (0.1 g Coomassie Brilliant Blue G-250 in 10 mL of 95% ethanol), followed by double-distilled H_2O up to 18 µL. The samples were loaded on a 4-15% Criterion TGX Precast gel (Bio-Rad) with NativeMark Unstained Protein Standard (Thermo Fisher Scientific) in a parallel lane and run in 1X Novex Tris-Glycine Native Running Buffer (Thermo Fisher Scientific) mixed with 0.1X NativePAGE Cathode Buffer Additive (Thermo Fisher Scientific) and then run at 12 W for 1 h at 4℃ using a Criterion Cell (Bio-Rad) according to the manufacturer's protocol.

Factor Xa Cleavage of the N-terminal MBP Tag. To prepare G2L4 RT for crystallization, the N-terminal MBP tag was removed by incubating 50 µg/mL MBP-G2L4 RT with 1 µg/mL of Factor Xa (New England Biolabs) in 20 mM Tris-HCl pH 7.5, 50 mM NaCl, 2 mM $CaCl_2$ at 4℃ overnight. Following cleavage, G2L4 RT was loaded onto a 5-mL HiTrap MBP HP column (Cytiva) at flow rate of 5 mL/min using an AKTA™ start FPLC (Cytiva). The flow-through was collected and then loaded onto a 5-mL HiTrap Heparin HP column (Cytiva) at flow rate of 5 mL/min, and the column was washed with five column volumes of buffer A. Bound proteins were eluted with a ten-column volume NaCl gradient using buffer A and buffer B (see above). Column fractions containing the RT protein were identified by SDS-PAGE, pooled, and protein concentration was measured by Bradford Protein assay kit (Bio-Rad) using the manufacturer's protocol. The pooled protein fractions were concentrated to 8-9 mg/mL in crystallization buffer containing 5 mM Tris-HCl pH 7.5, 50 mM NaCl, 5 mM DTT and 10% glycerol using an Amicon Ultra-15 (30k) concentrator (Millipore Sigma) at 4℃ and stored at -80℃ until used.

G2L4 RT Crystallization. For crystallization of G2L4 RT apoenzyme, a sitting drop mixture containing 1 µL of 10 mg/mL G2L4 RT without an MBP tag was incubated at 22℃ with an equal

volume of crystallization buffer containing 0.1 M Bis-Tris pH 5.5, 0.2 M ammonium acetate and 25% polyethyleneglycol 3350 in an Intelli-Plate 96-2 Low Profile plate (Hampton Research). Crystals were observed after 5-8 days and harvested after 1 month. For co-crystallization, G2L4 RT without an MBP tag was prepared in crystallization buffer and mixed with a 15-nt snapback DNA oligonucleotide (IDT; Table S2) at a 1:1.2 molar ratio of protein to ligand in a final volume of 100 µL. The mixture also included 20 mM $MgCl_2$, 1 mM $MnCl_2$, 2 mM dCTP (New England Biolabs), and 1 mM ddGTP (Millipore Sigma). After incubation at room temperature for 30 min, the mixture was set up in crystal trays. The final crystals were obtained in 0.2 M magnesium chloride hexahydrate, 0.1 M Bis-Tris pH 6.5, 25% polyethylene glycol 3350 (Hampton Research) at 25℃ after 5-8 days and were harvested after 16 days. Both apoenzyme G2L4 RT crystals and co-crystals of G2L4 RT with a snap-back DNA oligonucleotide, dCTP and ddGTP were mounted by looping directly from the crystallization drop and plunging into liquid nitrogen for cryo-protection.

G2L4 RT Apoenzyme and Snapback DNA Complex Data Collection, Analysis, and Structure Determination. Diffraction data for G2L4 RT apoenzyme were collected on beamline 5.0.1 of the Advanced Light Source (ALS) at 100K at the Lawrence Berkeley National Laboratory. Images were integrated using the XDS package (8) and scaled with Aimless (9). Initial structural determination was carried out using selenomethionine single-wavelength anomalous diffraction (SAD) phasing on 720 degrees of data from one crystal that diffracted to 2.9 Å and showed a weak anomalous signal to 4 Å. Native crystal diffraction data were also collected in the same beamline (Table S1). ShelxC/D followed by Autosol from the Phenix package (10) was used to generate a density-modified map that showed the three-helix bundle of the thumb and some clearly visible regions of the palm. Partial model building of the thumb and palm region was performed in the SAD density-modified map in Coot (11) and then refined in Refmac5 (12) and used as a molecular replacement model in Phaser for the higher quality 2.6-Å native dataset. Further rounds of model building and refinement were carried out using a combination of Buster (13) and Phenix, applying bulk solvent parameters, NCS restraints, TLS, and individual temperature factors. Diffraction data for G2L4 RT snapback complex were collected on beamline 5.0.2 of the Advanced Light Source (ALS) at 100K. X-ray diffraction data were processed to a resolution of 2.77 Å using HKL2000 (14) (Table S1). The molecular replacement solution for the complex structure was iteratively built using Coot with the Phenix refine package (15). Final refinement was completed using Refmac (12). The quality of the final refined structure was evaluated by MolProbity (16). Statistics for data collection and structure determination are shown in Table S1.

Structural Figures and Map Visualization. All structural figures were created using USCF ChimeraX (version 1.8) (17). Electron density maps (2Fo–Fc and Fo–Fc) were generated using phenix.maps in the Phenix software suite (18). The Fo-Fc map was contoured at 3 σ to highlight significant differences between the observed and modeled electron density. The 2Fo-Fc map was contoured at 1.5 σ to visualize the electron density, aiding in model refinement and validation. Positive densities confirmed the presence of modeled atoms, while absent or poorly contoured densities suggested model inaccuracies.

Differential scanning fluorimetry. A Roche Light Cycler® 480II was used for differential scanning fluorimetry (DSF) assays. SYPRO Orange dye (Thermo Fisher Scientific, 5,000x in DMSO) was used for fluorescence emission. The reaction was prepared in a total volume of 20 µL with a final protein concentration of 5 µM. The dilution buffer used was identical to the gel filtration buffer, consisting of 20 mM Tris-HCl pH 7.5, 50 mM NaCl, 0.1% % ß-mercaptoethanol, 10% glycerol. SYPRO Orange dye (5,000x stock solution) was added to the reaction mix at a final concentration of 1x. The mixture was placed in a 96-well plate designed for a real-time

PCR instrument (Life Technologies), thoroughly mixed, incubated for 10 min at room temperature, and then centrifuged at 2,000 × g for 5 min. The assay plate was placed into a Roche Light Cycler® 480II to obtain a pre-programmed experimental Melting Curve with target temperature set for 95℃, retention time set at continuous, and ramp rate at 0.04℃/second. The software analysis function was used to calculate the melting temperature (Tm) for each sample.

Size-Exclusion Chromatography
WT and mutant G2L4 RTs with or without an N-terminal MBP tag were purified as described above. The oligomeric state of the proteins was evaluated using HiLoad 16/600 Superdex 200 (Cytiva) and Superdex 75 prep grade columns (Cytiva) for proteins with or without an MBP tag, respectively. Concentrated protein samples were injected onto the columns using a Bio-Rad chromatography system. Elution was carried out at a flow rate of 0.8 mL/min with a running buffer comprised of 20 mM Tris-HCl pH 7.5, 50 mM NaCl, 0.1% β-mercaptoethanol, and 10% glycerol at 4°C. UV absorbance at 280 nm was monitored with a Bio-Rad detector to track the elution profiles. Molecular weights were calculated based on the calibration curve for elution volumes versus the logarithm of the molecular weight of Gel Filtration Standard (Bio-Rad). Fractions were collected for further analysis, and the molecular weights of the proteins in solution were determined using a previously established calibration curve to assess their oligomeric state. Following size-exclusion chromatography, proteins were concentrated to 8-9 mg/mL in a crystallization buffer containing 5 mM Tris-HCl pH 7.5, 50 mM NaCl, and 10% glycerol for subsequent crystallization experiments.

Primer Extension Assays. Biochemical assays were done using G2L4 RTs stabilized with an N-terminal MBP-tag. Primer extension assays were done by using a 50-nt DNA template oligonucleotide ending with an inverted 3' dT that blocks use for snapback DNA synthesis or terminal transferase activity (IDT; Table S2). The DNA template (1 µM) was pre-annealed to 200 µM of a 5-nt DNA primer in 100 µL of TE (10 mM Tris-HCl pH 7.5, 1 mM EDTA) by heating to 95°C for 3 min followed by cooling to 25°C at 0.1°C /min in a T100 thermal cycler (Bio-Rad). The assays were performed in 80 µL of reaction medium containing 500 nM WT or mutant G2L4 RTs, 250 nM template-primer complex, 20 mM Tris-HCl pH 7.5, 20 mM NaCl, and 10 mM $MgCl_2$ with or without 1 mM $MnCl_2$. After pre-incubating the RT with the annealed template-primer substrate for 30 min at room temperature, the reactions were initiated by adding 1 mM dNTPs (1 mM each of dATP, dCTP, dGTP and dTTP) plus 1 µCi [α-^{32}P]-dTTP (3,000 Ci/mmol; Revvity) and incubated at 37°C for times up to 180 min. For time courses, 10-µL aliquots were taken at each time point and quenched by adding 2 µL of 6X stop solution (25 mM EDTA, 0.5 U/µL Proteinase K (New England Biolabs) and incubating for 15 min at 37°C. The samples were then mixed with an equal volume of 2X RNA loading dye (95% formamide, 0.02% SDS, 0.02% bromophenol blue, 0.01% xylene cyanol, 1 mM EDTA) and analyzed by electrophoresis in a 20% polyacrylamide gel with TBE (89 mM Tris, 89 mM borate and 2 mM EDTA) buffer against 5'-[^{32}P]-labeled synthetic ssDNA size markers. The gel was dried and scanned using a phosphorimager (Typhoon FLA 9500; GE Healthcare) and processed with ImageJ (19). The amount of labeled dTTP incorporated into the product was quantified using ImageQuant TL 10.2. To account for differences in primer extension product sizes, the label amount was normalized by multiplying the dTTP concentration (1 mM) and dividing by the number of T residues (22 bases) per extension product. This provided the concentration of extended product, which was then plotted relative to the template concentration (250 nM). Time course data were fit to a first-order rate equation using Prism 10.0 to obtain k_{obs} and amplitude values. For slow reactions without a clear end point, the reaction amplitude was fit to match the average end point of parallel reactions that reached clear end points. Amplitude values obtained in this way are indicated in parenthesis in tables next to the plots.

Snapback DNA Synthesis Assays. The substrate for snapback DNA synthesis assays was a 50-nt DNA oligonucleotides with an unblocked 3' OH end (Table S2). The assays were done as time courses by pre-incubating 10 nM 5'-^{32}P -labeled 50-nt DNA oligonucleotide with 500 nM enzyme in 80 µL of the reaction medium containing 20 mM Tris-HCl pH 7.5, 20 mM NaCl, 10 mM $MgCl_2$ and with or without 1 mM $MnCl_2$ for 30 min at room temperature. Reactions were initiated by adding 1 mM dNTPs (an equimolar mix of 1 mM dATP, dCTP, dGTP, and dTTP). 10-µL aliquots were taken at each time point up to 180 min, quenched with 2 µL of 6X stop solution, and analyzed by electrophoresis in a non-denaturing 12% polyacrylamide gel with TBE (89 mM Tris, 89 mM borate and 2 mM EDTA) buffer against double-stranded DNA size markers, a 5'-labeled Low Molecular Weight DNA Ladder (New England Biolabs), The gel was dried, scanned with a phosphorimager (Typhoon FLA 9500; GE Healthcare), and processed with ImageJ. Products were quantified with ImageQuant TL 10.2, and data were analyzed as described above for primer extension assays.

Microhomology-Mediated End-Joining Assays. MMEJ assays was performed as described (1) with minor modifications. The MMEJ assays were done using partially double-stranded DNA substrates consisting of a 5'-labeled 53-nt oligonucleotide (D1) ending with a 4-nt microhomology annealed to an unlabeled complementary 39-nt DNA oligonucleotide with a 3' inverted dT blocking group (D2) leaving a 15-nt single-stranded 3' overhang. To anneal the complementary strands of the MMEJ substrate, 10 µM D1 and 20 µM D2 oligonucleotides in 100 µL TE buffer were heated to 95°C for 3 min and cooled at 0.1°C/min to 25°C in a T100 thermal cycler (Bio-Rad). In Figures, the annealed products are denoted D1/D2 and D1'/D2'. Reactions were done using 250 nM of the annealed substrate preincubated with 500 nM WT or mutant G2L4 RT in the reaction medium containing 20 mM Tris-HCl pH 7.5, 20 mM NaCl, and 10 mM $MgCl_2$ with or without 1 mM $MnCl_2$ for 30 min at room temperature. The reactions were initiated by adding 1 mM dNTPs (an equimolar mix of 1mM dATP, dCTP, dGTP, and dTTP), incubated at 37°C for up to 180 min, and quenched as described for primer extension assays. Products were analyzed by electrophoresis in a non-denaturing 12% polyacrylamide gel with TBE (89 mM Tris, 89 mM borate and 2 mM EDTA) buffer against double-stranded DNA size markers (5'-labeled Low Molecular Weight DNA Ladder; New England Biolabs). The gel was scanned with a phosphorimager (Typhoon FLA 9500; GE Healthcare) and processed using ImageJ. Products were quantified with ImageQuant TL 10.2, and data were analyzed as described for primer extension assays.

Terminal Transferase Assays. Terminal transferase assays were done using the same 50-nt DNA oligonucleotides used as templates in primer extension assays but 5'-^{32}P-labeled and without a 3'-blocking group (Table S2). WT or mutant G2L4 RT (500 nM) was pre-incubated with 5'-labeled oligonucleotide substrate (10 nM) in reaction medium containing 20 mM NaCl, 10 mM $MgCl_2$, and 20 mM Tris-HCl pH 7.5 with or without 1 mM $MnCl_2$ for 30 min at room temperature. The reaction was initiated by adding 1 mM of a single dNTP (dATP, dCTP, dGTP, or dTTP), incubated at 37°C for times indicated for individual experiments, and then quenched as described for primer extension assays. Products were analyzed in a denaturing 6% polyacrylamide gel with TBE (89 mM Tris, 89 mM borate and 2 mM EDTA) buffer against a 5'-labeled RiboRuler Low Range RNA Ladder (Thermo Fisher Scientific) as size markers The gel was dried, scanned with a phosphorimager (Typhoon FLA 9500; GE Healthcare), and processed using ImageJ. Quantification was done with ImageQuant TL 10.2, and data were analyzed as described for primer extension assays.

Structural Modeling for the Potential G2L4 RT DSBR mechanism in Fig. 6. Unwound DNA from the superfamily 2 helicase Hel308 complex (PDB ID: 2P6R) (20) was used as a structural basis to model the MMEJ substrate in the G2L4 RT-mediated DSBR pathway. The partially

unwound DNA in this structure consisted of a 5 to 3′ 25-mer strand and a 3′ to 5′ 15-mer strand. Using Coot, the last two unpaired nucleotides of the 15-mer strand were trimmed for the model to generate a partial duplex with a 12-nt single-stranded 3'-overhang. For the MMEJ mechanism model in Fig. 6, one copy was modified by shortening the 3′ single-stranded overhang to 10 nucleotides and changing the last five nucleotides to AACCG. This modified strand was then superimposed onto the primer strand of a snapback substrate using the Coots LSQ superimposition function. Another copy of the duplex structure was altered by generating four additional nucleotides along the 3′ single-stranded overhang and mutating the last six nucleotides to GCGGTT. This modified strand was superimposed onto the template strand of a snapback substrate using the same LSQ superimposition function. Following alignment, overlapping nucleotides at the 3′ ends of the modified strands were removed to eliminate redundancy. The three molecules were then merged, and Coot's Real Space Refinement function was applied to connect the original substrate and the additional partial duplex, resulting in the generation of two new chains: one 29 nt in length, corresponding to the modified template strand, and the other 23 nt in length, corresponding to the modified primer strand. The resulting structural model was subsequently prepared using the Protein Preparation Workflow in Maestro (Schrödinger Suite version 2023-1) (21) with default parameters. This workflow facilitated energy minimization to refine the geometry of the model.

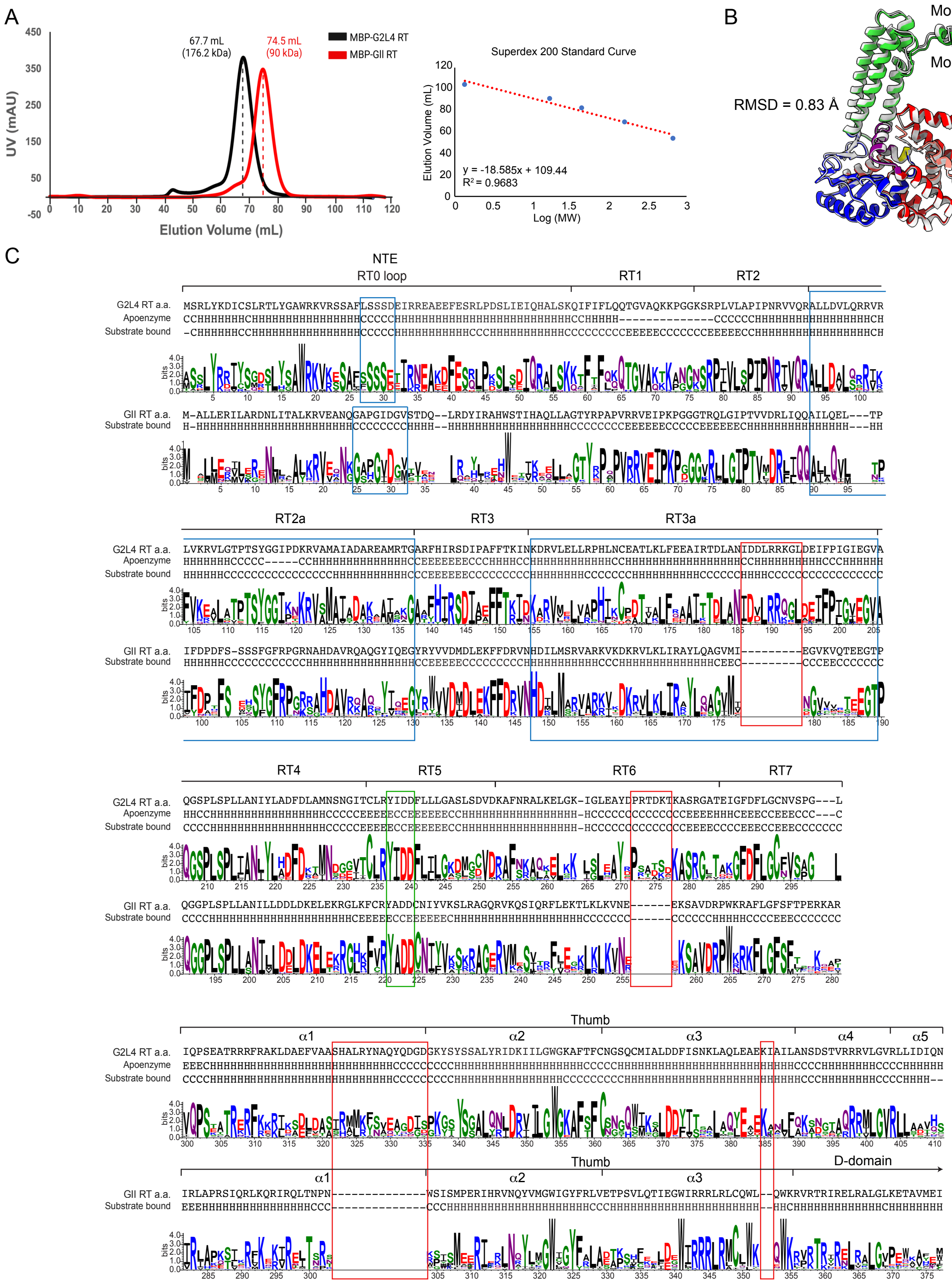
A
67.7 mL
(176.2 kDa)
74.5 mL
(90 kDa)
MBP-G2L4 RT
MBP-GII RT
UV (mAU)
Elution Volume (mL)
Superdex 200 Standard Curve
Elution Volume (mL)
Log (MW)
y = -18.585x + 109.44
R² = 0.9683
B
Monomer B
Monomer A
RMSD = 0.83 Å
C
NTE
RT0 loop
RT1
RT2
RT2a
RT3
RT3a
RT4
RT5
RT6
RT7
Thumb
D-domain
G2L4 RT a.a.
Apoenzyme
Substrate bound
GII RT a.a.
Substrate bound
bits

Fig. S1. Characteristics and comparisons of G2L4 and GII RTs. (*A*) Superdex 200 size-exclusion chromatography profiles of purified MBP-tagged G2L4 RT (black) and GII RT (red). Vertical dashed lines indicate peak elution volumes. Molecular weights of the proteins based on the peak elution volumes were calculated by using the calibration curve for $\log_{10}$ molecular weight (MW) of Superdex 200 protein standards shown to the right. (*B*) Superimposition of the two monomer subunits of G2L4 RT in the X-ray crystal structure of apoenzyme dimers. Regions of monomer A are color-coded as in Fig. 1*A* and monomer B is colored gray. (*C*) Sequence alignment of G2L4 and GII RTs done by ClusterW via Jalview (4, 5). WebLogos are based on 130 G2L4 RTs and 500 GII RTs searched by BLASTP (3) for ≥50% Identity of amino acids sequence and aligned by ClustalW. Sequence motifs found in all RTs (RT1-7) and NTE/RT0 loop, RT2a, and RT3a found in non-LTR-retroelement RTs are delineated above the alignments with the latter highlighted in blue boxes. The YxDD motif at the RT active site is boxed in green. Red boxes indicate insertions in G2L4 RT relative to GII RT. S412 of G2L4 RT is derived from the expression plasmid. Amino acid residues in α-helical (H), random coil (C), and β-sheet (E) regions in the indicated crystal structures are indicated below the amino acid sequence. α1 through α5 are thumb domain α-helices in G2L4 RT labeled in Fig. 1*B*. The last two amino acids of G2L4 RT were not visible in the substrate-bound active structure.

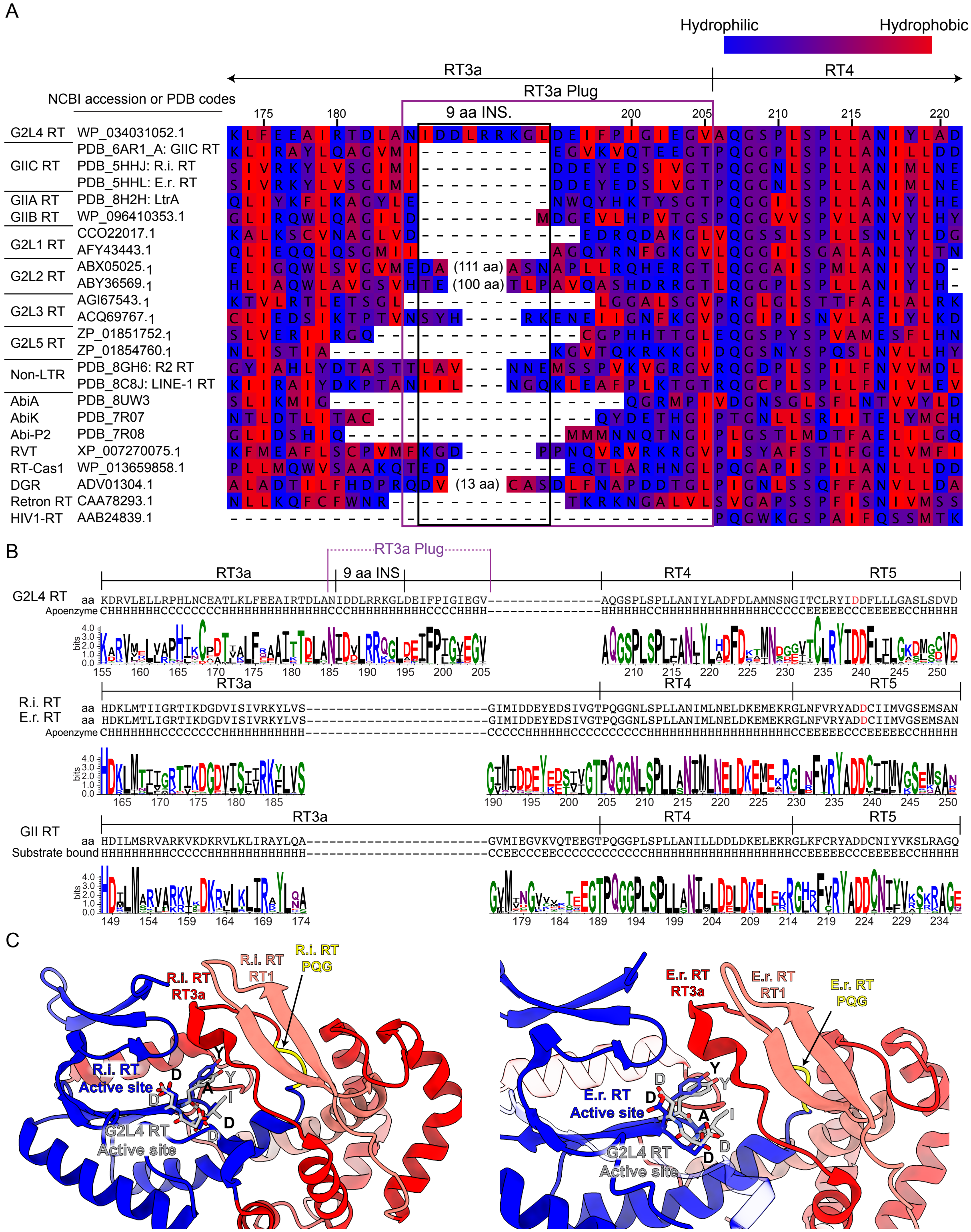

A
Hydrophilic
Hydrophobic
RT3a
RT4
RT3a Plug
9 aa INS.
NCBI accession or PDB codes
G2L4 RT WP_034031052.1
PDB_6AR1_A: GIIC RT
GIIC RT PDB_5HHJ: R.i. RT
PDB_5HHL: E.r. RT
GIIA RT PDB_8H2H: LtrA
GIIB RT WP_096410353.1
CCO22017.1
G2L1 RT AFY43443.1
ABX05025.1
G2L2 RT ABY36569.1
AGI67543.1
G2L3 RT ACQ69767.1
ZP_01851752.1
G2L5 RT ZP_01854760.1
PDB_8GH6: R2 RT
Non-LTR PDB_8C8J: LINE-1 RT
AbiA PDB_8UW3
AbiK PDB_7R07
Abi-P2 PDB_7R08
RVT XP_007270075.1
RT-Cas1 WP_013659858.1
DGR ADV01304.1
Retron RT CAA78293.1
HIV1-RT AAB24839.1
(111 aa)
(100 aa)
(13 aa)
B
RT3a Plug
9 aa INS
RT5
G2L4 RT
aa KDRVLELLRPHLNCEATLKLFEEAIRTDLANIDDLRRKGLDEIFPIGIEGV---------------AQGSPLSPLLANIYLADFDLAMNSNGITCLRYIDDFLLLGASLSDVD
Apoenzyme
R.i. RT
E.r. RT
aa HDKLMTIIGRTIKDGDVISIVRKYLVS------------------------GIMIDDEYEDSIVGTPQGGNLSPLLANIMLNELDKEMEKRGLNFVRYADDCIIMVGSEMSAN
aa HDKLMTLIGRTIKDGDVISIVRKYLVS------------------------GIMIDDEYEDSIVGTPQGGNLSPLLANIMLNELDKEMEKRGLNFVRYADDCIIMVGSEMSAN
GII RT
aa HDILMSRVARKVKDKRVLKLIRAYLQA------------------------GVMIEGVKVQTEEGTPQGGPLSPLLANILLDDLDKELEKRGLKFCRYADDCNIYVKSLRAGQ
Substrate bound
bits
C
R.i. RT RT3a
R.i. RT RT1
R.i. RT PQG
R.i. RT Active site
G2L4 RT Active site
E.r. RT RT3a
E.r. RT RT1
E.r. RT PQG
E.r. RT Active site

Fig. S2. Comparison of RT3a and RT4 regions of G2L4 RT to those of other RTs. (*A*) Sequence alignment of the RT3a/RT4 region of G2L4 RT (top) with those of other RTs (NCBI accession numbers or PDB codes to the right of the protein names) performed by T-Coffee via Jalview (4, 7). Other RTs include group IIC, IIA, and IIB intron RTs; bacterial chromosomally encoded group II intron-like 1, 2, 3, and 5 RTs; non-LTR-retrotransposon RTs; other chromosomally encoded bacterial RTs, including RVT, which is also found in some eukaryotes; and retrovirus HIV-1 RT. The G2L4 RT3a plug region is highlighted in a purple box, with the 9-aa insertion within the G2L4 RT3a plug highlighted in a black box. Amino acid residues in boxes are colored-coded by their hydropathic character as charged (blue), polar (purple), or hydrophobic (red) (22). The numbers at the top indicate the positions of amino acids in the G2L4 RT sequence. Numbers in parentheses in gaps in the alignments for some RTs indicate the number of additional amino acids. (*B*) Amino acids sequence alignments and WebLogos comparing the RT3a, RT4, and RT5 regions of G2L4 RTs to those of *Roseburia intestinalis* (R.i) and *Eubacterium rectale* (E.r.) group IIC intron RTs, which lack an RT3a plug. The RT3a plug and its 9-aa insertion in G2L4 RT are highlighted in the alignment to illustrate their evolutionary conserved amino acid sequence in 130 different G2L4 RTs. Amino acid residues in α-helical (H), random coil (C), and β-sheet (E) regions in crystal structures are indicated below the amino acid sequences. (*C*) Superimposition of crystal structures of the fingers and palm regions of the R.i. (PDB: 5HHJ; left) and E.r. (PDB: 5HHL; right) RTs (23) colored as in Fig. 1*B* with that of G2L4 RT (gray). Active-site residues are colored blue in R.i. and E.r. RTs and gray in G2L4 RT with oxygens colored red. The arrows point to the partially hidden PQG motifs (yellow) at the beginning of RT4 of the R.i. and E.r. RTs.

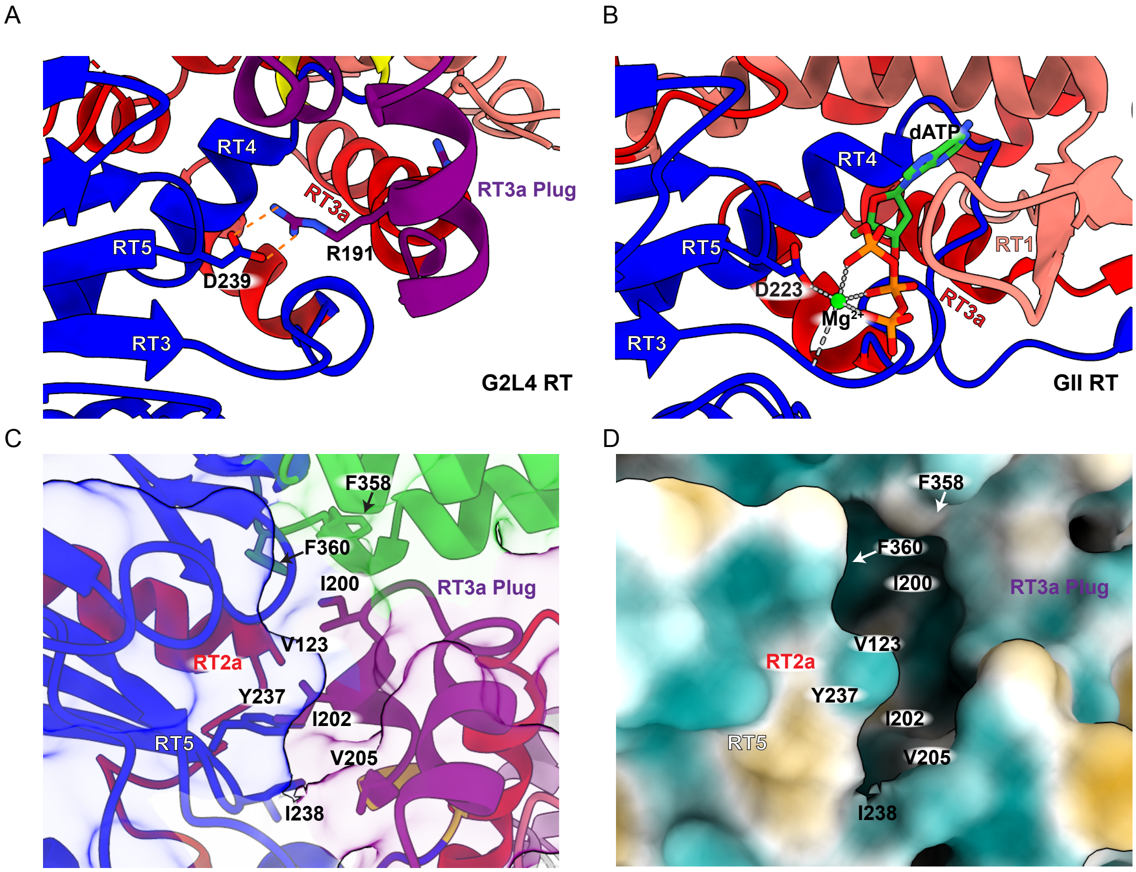

Fig. S3. RT3a plug interactions with the active site and thumb domain of G2L4 RT. (*A* and *B*) Close-up views of the active-site region of G2L4 RT apoenzyme compared to that of the active structure of GII RT (PDB: 6AR1). Key residues are shown as sticks colored by region (palm, blue; fingers, salmon; RT3a red; RT3a plug, purple with oxygens, red and nitrogens, blue). In panel *A*, orange dashed lines show salt bridges of the active-site aspartate (D239) with R191 in the RT3a plug of G2L4 RT apoenzyme. In panel *B*, gray dashed lines shown octahedral coordination of a GII RT active-site Mg^{2+} (green sphere) with the corresponding aspartate (D223) and incoming dATP (stick with carbons, green; nitrogens, blue; phosphorus, orange; and oxygens, red). (*C)* Hydrophobic interactions between the RT3a plug and the active site and thumb domain of G2L4 RT. Side chains of hydrophobic residues from the RT3a plug, active site, and thumb domain are shown as sticks colored by region (palm, blue; thumb, green; RT2a, red; RT3a plug, purple, with colors muted by surface overlays). The surface of the protein is represented by lightly shaded overlays colored by region and demarcated by thin lines. (*D*) A non-transparent hydrophobicity surface view of the panel *C* region of G2L4 RT with hydrophobic regions, beige and hydrophilic regions, teal. Areas of darkness in panel *D* regions are due to shadowing.

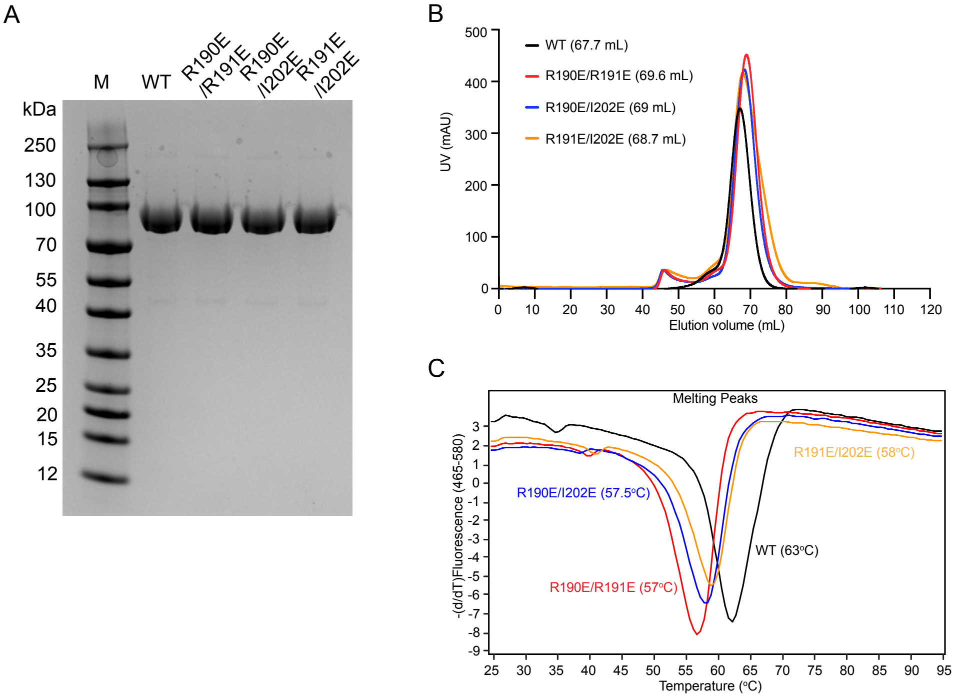

Fig. S4. Biochemical properties of WT and RT3a mutant G2L4 RTs. (*A)* Coomassie blue-stained NuPAGE 4-12% Bis-Tris gel of WT and RT3a mutant G2L4 RTs used in biochemical assays. Proteins were expressed with a N-terminal maltose-binding protein (MBP) tag and purified as described in Materials and Methods. The numbers to the left of the gel indicate molecular weights of a Color Prestained Broad Range (10-250 kDa) protein ladder (New England Biolabs) in the left lane. (*B*) Size-exclusion chromatography of purified WT and RT3a mutant MBP-tagged G2L4 RTs. Elution profiles of different proteins are color-coded as indicated in the Figure. (*C*) Differential scanning fluorimetry (DSF) of WT and RT3a mutant MBP-tagged G2L4 RTs. Melting profiles of different protein are color-coded as in panel *B*. The plots show the derivative of fluorescence intensity as a function of temperature, revealing transitions or melting points indicative of stability shifts.

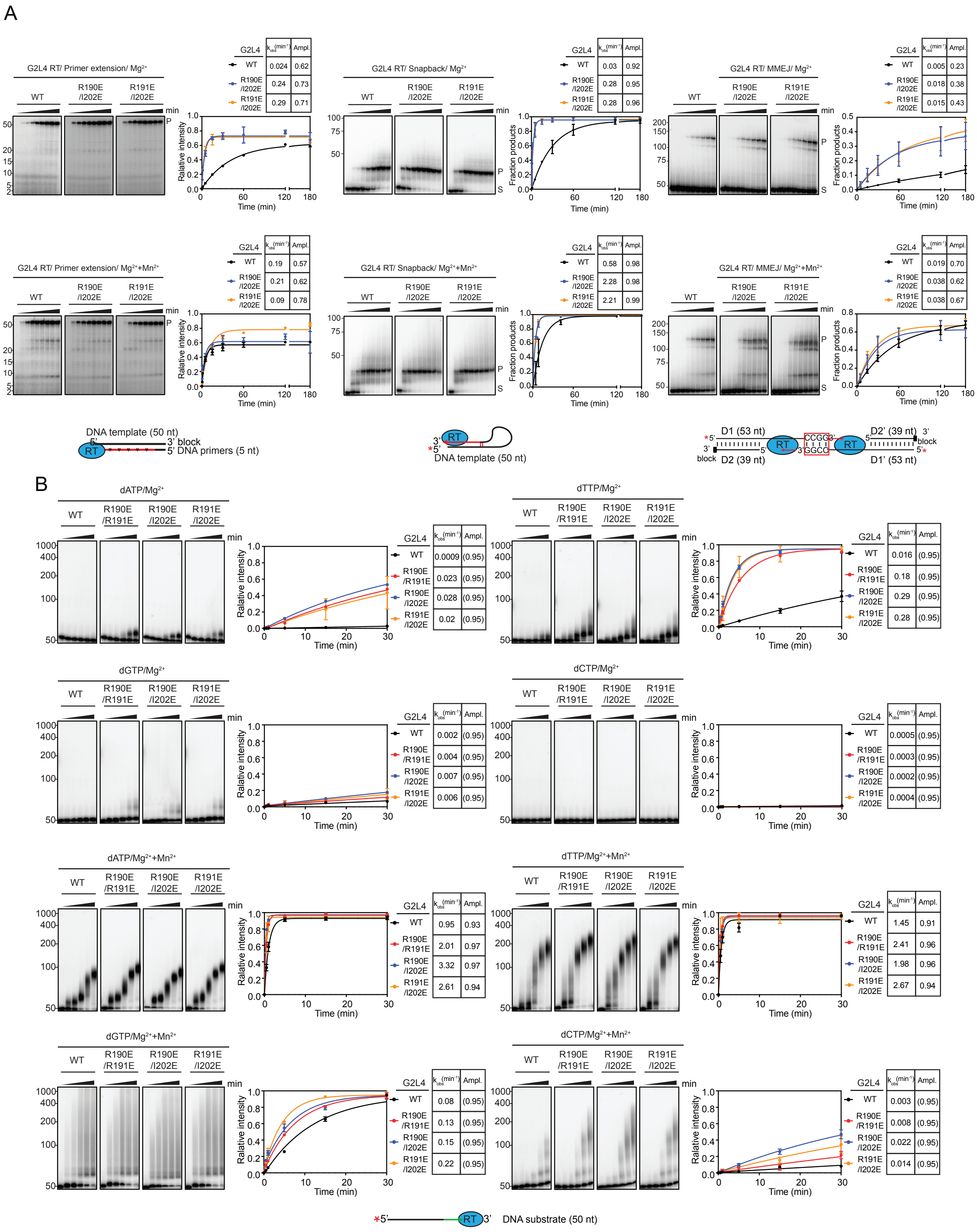
A
G2L4 RT/ Primer extension/ Mg2+
WT
R190E /I202E
R191E /I202E
min
P
Relative intensity
Time (min)
G2L4
kobs(min-1)
Ampl.
WT 0.024 0.62
R190E /I202E 0.24 0.73
R191E /I202E 0.29 0.71
G2L4 RT/ Snapback/ Mg2+
Fraction products
WT 0.03 0.92
R190E /I202E 0.28 0.95
R191E /I202E 0.28 0.96
G2L4 RT/ MMEJ/ Mg2+
WT 0.005 0.23
R190E /I202E 0.018 0.38
R191E /I202E 0.015 0.43
G2L4 RT/ Primer extension/ Mg2++Mn2+
WT 0.19 0.57
R190E /I202E 0.21 0.62
R191E /I202E 0.09 0.78
G2L4 RT/ Snapback/ Mg2++Mn2+
WT 0.58 0.98
R190E /I202E 2.28 0.98
R191E /I202E 2.21 0.99
G2L4 RT/ MMEJ/ Mg2++Mn2+
WT 0.019 0.70
R190E /I202E 0.038 0.62
R191E /I202E 0.038 0.67
DNA template (50 nt)
3' block
5' DNA primers (5 nt)
RT
DNA template (50 nt)
D1 (53 nt)
D2' (39 nt)
block
D2 (39 nt)
D1' (53 nt)
CCGG
GGCC
B
dATP/Mg2+
R190E /R191E
Ralative intensity
WT 0.0009 (0.95)
R190E /R191E 0.023 (0.95)
R190E /I202E 0.028 (0.95)
R191E /I202E 0.02 (0.95)
dTTP/Mg2+
WT 0.016 (0.95)
R190E /R191E 0.18 (0.95)
R190E /I202E 0.29 (0.95)
R191E /I202E 0.28 (0.95)
dGTP/Mg2+
WT 0.002 (0.95)
R190E /R191E 0.004 (0.95)
R190E /I202E 0.007 (0.95)
R191E /I202E 0.006 (0.95)
dCTP/Mg2+
WT 0.0005 (0.95)
R190E /R191E 0.0003 (0.95)
R190E /I202E 0.0002 (0.95)
R191E /I202E 0.0004 (0.95)
dATP/Mg2++Mn2+
WT 0.95 0.93
R190E /R191E 2.01 0.97
R190E /I202E 3.32 0.97
R191E /I202E 2.61 0.94
dTTP/Mg2++Mn2+
WT 1.45 0.91
R190E /R191E 2.41 0.96
R190E /I202E 1.98 0.96
R191E /I202E 2.67 0.94
dGTP/Mg2++Mn2+
WT 0.08 (0.95)
R190E /R191E 0.13 (0.95)
R190E /I202E 0.15 (0.95)
R191E /I202E 0.22 (0.95)
dCTP/Mg2++Mn2+
WT 0.003 (0.95)
R190E /R191E 0.008 (0.95)
R190E /I202E 0.022 (0.95)
R191E /I202E 0.014 (0.95)
DNA substrate (50 nt)

Fig. S5. Additional biochemical assays of WT and RT3a plug mutant G2L4 RTs. (A) Primer extension, snapback DNA synthesis, and MMEJ assays for mutants R190E/I202E and R191E/I202E compared to WT G2L4 RT. Reactions were done as time courses with MBP-tagged proteins, as described for Fig. 2*D*. (B) Terminal transfers assays for mutants R190E/R191E, R190E/I202E, and R191E/I202E compared to WT G2L4 RT. Reactions with MBP-tagged proteins were done as time courses with a 5'-^{32}P-labeled 50-nt DNA substrate without a 3'-blocking group. Reactions were initiated by adding 1 mM of a single dNTP (dATP, dCTP, dGTP, and dTTP) and incubated at 37°C for times up to 30 min in reaction medium containing 10 mM Mg^{2+} in the absence (top two row) or presence (bottom two rows) of 1 mM Mn^{2+}. The numbers to the left of the gel indicate the positions of 5'-labeled RiboRuler Low Range RNA Ladder size markers run in a parallel lane. The plots to the right of the gels show the average values and variance for two repeats of the experiment. Tables to the right of the plots show the rate constants (k_{obs}) and amplitudes (Ampl.) of labeled products >50 nt obtained by fitting to a first-order rate equation. Ampl. values in parentheses represent fixed amplitudes for reactions that did not reach an end point based on the average Ampl. value for those that reached a clear end point during the experiment. The red asterisk in the schematic at the bottom indicates a 5'-^{32}P-label.

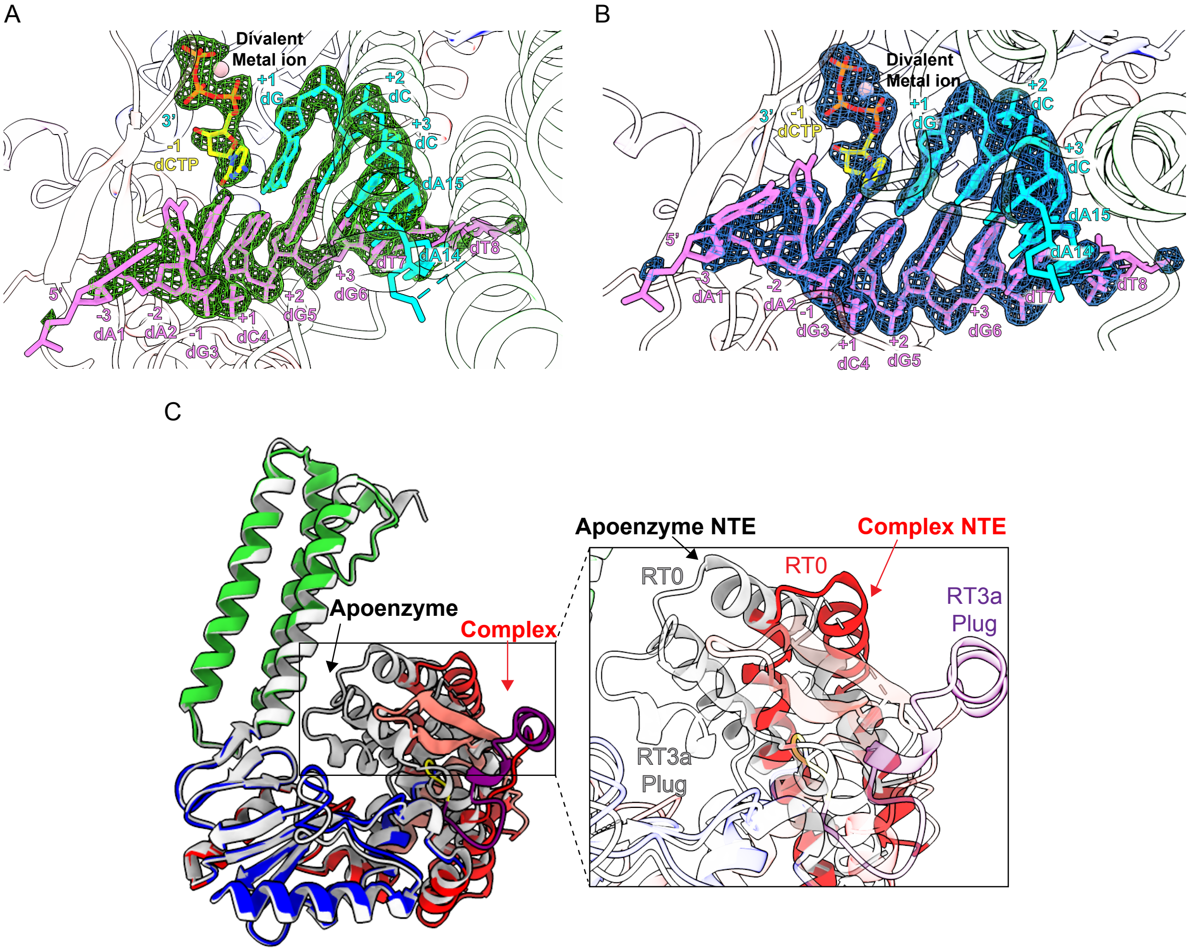

Fig. S6. Fo-Fc map and confirmatory 2Fo-Fc map of G2L4 RT complex with the bound snapback DNA substate and incoming dNTP. (*A*) Fo-Fc map calculated by subtracting the calculated electron density (Fc) from the observed electron density (Fo) for G2L4 RT to identify differences due to binding of the snapback DNA substrates. Positive density of the electron cloud is shown as green mesh contoured at a level of 3 sigma (σ). Regions of the encompassed snapback DNA substrate are colored as in panel A, and an incoming dCTP is shown as a stick (carbons, yellow; nitrogens, blue; phosphorus, orange; oxygens, red). The model showed positive density (green) in areas where an initial model lacked atoms, guiding the fit the snapback DNA in the correct position. (*B)* 2Fo-Fc map calculated by doubling the observed electron density (Fo) and subtracting the calculated electron density (Fc) to provide an estimate of the fitting of the electron density of the active site of G2L4 RT with bound substrate. The electron density is shown as blue mesh contoured to a level of 1.5 sigma (σ), and nucleotides are numbered and colored as in Fig. 3*A*. The 2Fo-Fc map confirmed that the newly placed DNA atom fit well into the overall electron density in a way consistent with experimental data. (*C*) Superimposition of a G2L4 RT monomer in snapback DNA complex (regions colored as in Fig. 1*A*) aligned at the palm with a monomer of the G2L4 RT apoenzyme (gray). The right panel close-up highlights the shift in position of the NTE/RT0 loop in the snapback DNA complex structure compared to that in the apoenzyme structure.

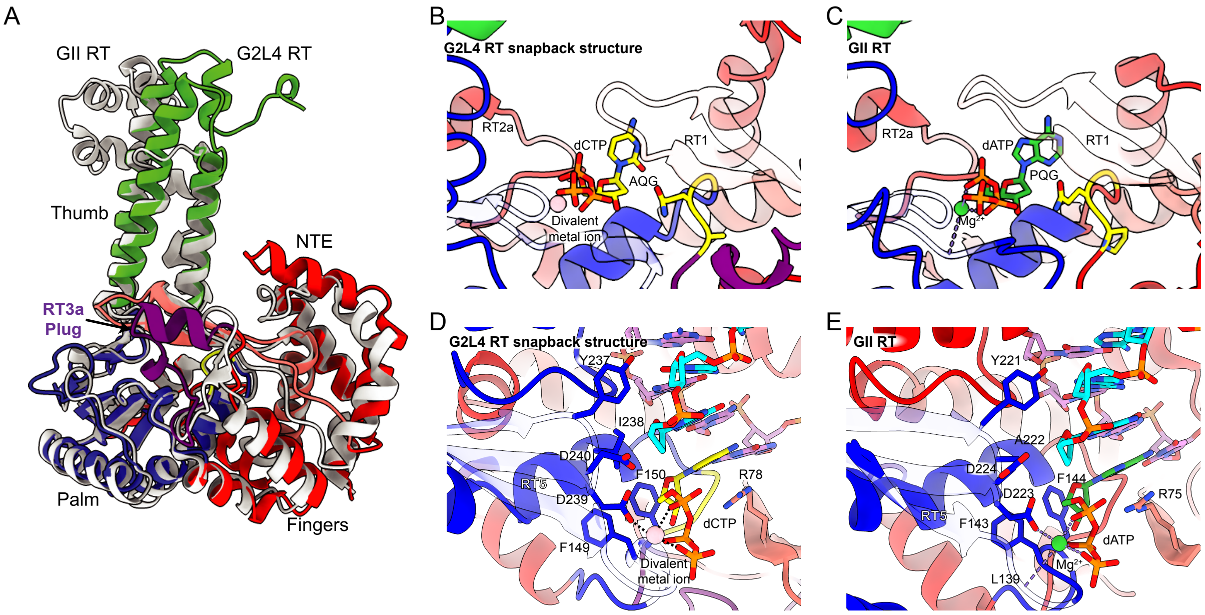

Fig. S7. Comparison of active structures of G2L4 and GII RTs. (*A*) Superimposition of the active conformations of G2L4 RT and GII RT. Different regions of G2L4 RT are color-coded as in Fig. 1, and GII RT is colored gray. (*B* and *C*) Close-ups comparing the active-site region of G2L4 RT in the snapback structure to that in the active structure of GII RT (PDB: 6AR1). Different regions of the proteins are colored as in Fig. 1*A* with AQG/PQG motifs yellow, divalent metal ion in G2L4 RT pink, and Mg^{2+} ion in GII RT green. dCTP in panel *B* is shown as a stick with carbons, yellow; nitrogens, blue; phosphorus, orange; oxygens, red, and dATP in panel *C* is shown as a stick with carbons, yellow and other atoms colored as in panel *B*. The AQG/PQG residues are shown as yellow sticks with nitrogen atoms blue and oxygen atoms red. (*D* and *E*) Close-ups comparing active site regions of G2L4 RT snapback structure and active structure GII RT with key residues in the DNA substrates shown as sticks (template strand violet and primer strand cyan) and other features colored as in panels C and D.

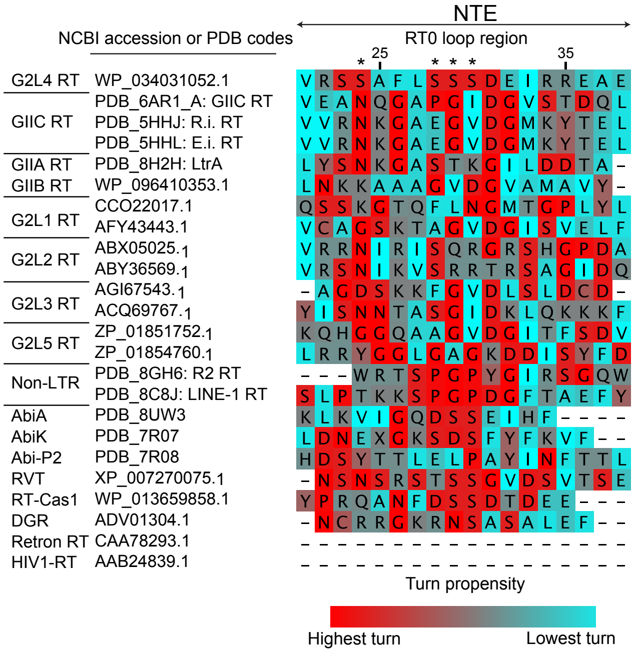

Fig. S8. Sequence alignments of the NTE/RT0 loop of G2L4 RT compared to other RTs. (*A*) Multiple sequence alignments of the NTE/RT0 loop region of G2L4 RT (top) with those of other RTs (NCBI accession numbers or PDB codes to the right of protein names) performed by T-Coffee via Jalview (4, 7). The color scheme of amino acids boxes is based on their Chou-Fasman two turn propensity (red, highest turn propensity; cyan, lowest turn propensity) (25). The numbers at the top indicate the positions in the G2L4 RT sequence, and the asterisks at the top indicate conserved serine residues in G2L4 RTs.

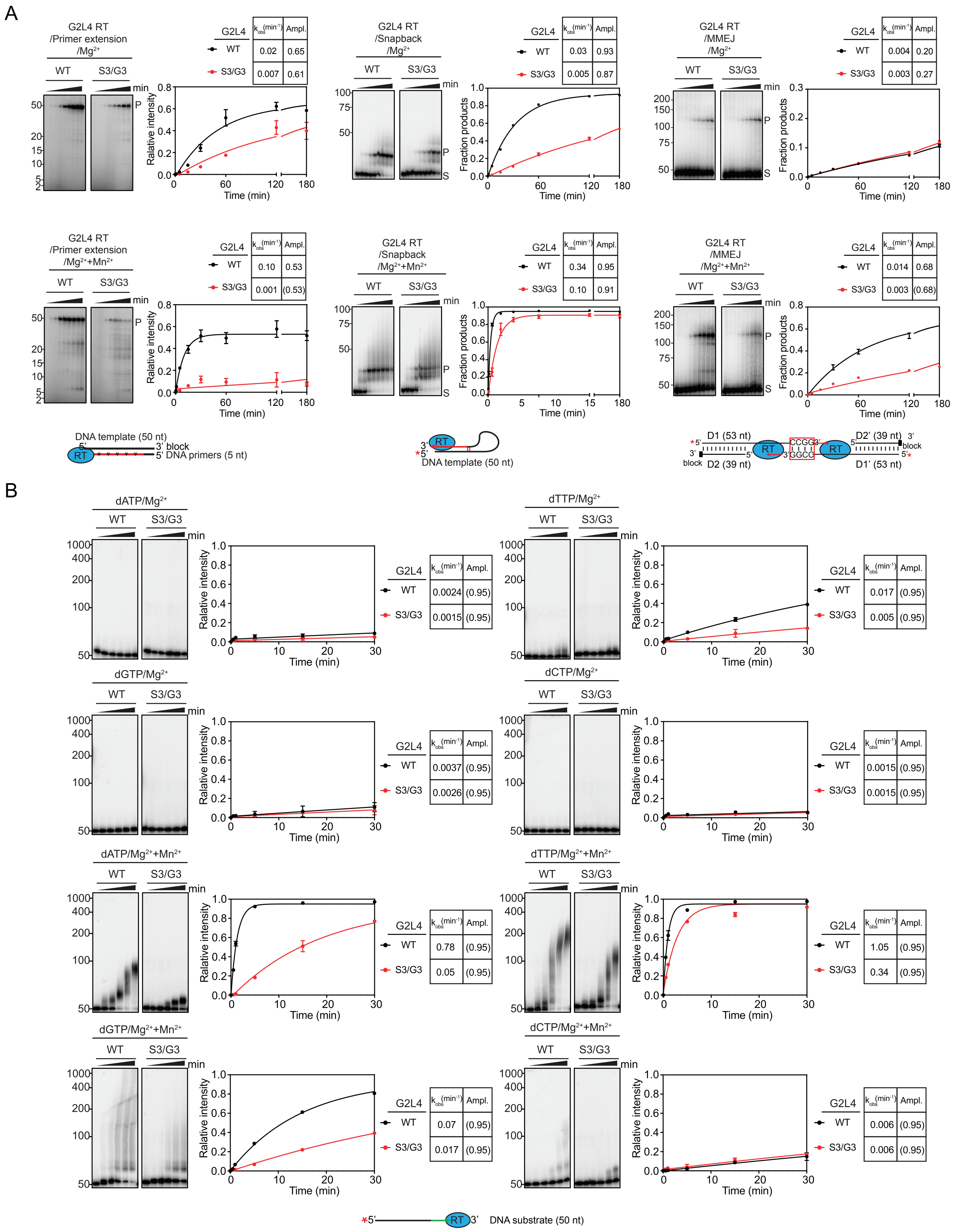
A
G2L4 RT /Primer extension /Mg2+
WT S3/G3 min
P
Ralative intensity
Time (min)
G2L4 kobs(min-1) Ampl.
WT 0.02 0.65
S3/G3 0.007 0.61
G2L4 RT /Snapback /Mg2+
Fraction products
G2L4 kobs(min-1) Ampl.
WT 0.03 0.93
S3/G3 0.005 0.87
G2L4 RT /MMEJ /Mg2+
G2L4 kobs(min-1) Ampl.
WT 0.004 0.20
S3/G3 0.003 0.27
G2L4 RT /Primer extension /Mg2++Mn2+
G2L4 kobs(min-1) Ampl.
WT 0.10 0.53
S3/G3 0.001 (0.53)
G2L4 RT /Snapback /Mg2++Mn2+
G2L4 kobs(min-1) Ampl.
WT 0.34 0.95
S3/G3 0.10 0.91
G2L4 RT /MMEJ /Mg2++Mn2+
G2L4 kobs(min-1) Ampl.
WT 0.014 0.68
S3/G3 0.003 (0.68)
DNA template (50 nt)
3' block
5' DNA primers (5 nt)
RT
DNA template (50 nt)
D1 (53 nt)
D2 (39 nt)
D2' (39 nt)
D1' (53 nt)
block
CCGG
GGCC
B
dATP/Mg2+
G2L4 kobs(min-1) Ampl.
WT 0.0024 (0.95)
S3/G3 0.0015 (0.95)
dTTP/Mg2+
G2L4 kobs(min-1) Ampl.
WT 0.017 (0.95)
S3/G3 0.005 (0.95)
dGTP/Mg2+
G2L4 kobs(min-1) Ampl.
WT 0.0037 (0.95)
S3/G3 0.0026 (0.95)
dCTP/Mg2+
G2L4 kobs(min-1) Ampl.
WT 0.0015 (0.95)
S3/G3 0.0015 (0.95)
dATP/Mg2++Mn2+
G2L4 kobs(min-1) Ampl.
WT 0.78 (0.95)
S3/G3 0.05 (0.95)
dTTP/Mg2++Mn2+
G2L4 kobs(min-1) Ampl.
WT 1.05 (0.95)
S3/G3 0.34 (0.95)
dGTP/Mg2++Mn2+
G2L4 kobs(min-1) Ampl.
WT 0.07 (0.95)
S3/G3 0.017 (0.95)
dCTP/Mg2++Mn2+
G2L4 kobs(min-1) Ampl.
WT 0.006 (0.95)
S3/G3 0.006 (0.95)
DNA substrate (50 nt)

Fig. S9. Biochemical assays of WT and S3/G3 mutant G2L4 RTs. (*A*) Primer extension, snapback DNA synthesis, and MMEJ assays assay with 10 mM Mg^{2+} (top row) or 10 mM Mg^{2+} plus 1 mM Mn^{2+} (bottom row). Reactions were done with MBP-tagged proteins, as described in the legend of Fig. 2*D* and Materials and Methods. The plots show the average values and variance for two repeats of the experiment. Red asterisks in schematics indicate ^{32}P-label. (*B*) Terminal transferase assays of MBP-tagged WT and S3/G3 mutant G2L4 RTs in the presence of 10 mM Mg^{2+} (top two rows) or 10 mM Mg^{+2} + 1 mM Mn^{2+} (bottom two rows) using a 5'-labeled 50-nt DNA substrate, as described in Fig. S5B and Materials and Methods. Reactions were initiated by adding 1 mM of a single dNTP (dATP, dCTP, dGTP, and dTTP) and incubated at 37°C for times up to 30 min. The numbers to the left of the gels indicate the positions of 5'-labeled RiboRuler Low Range RNA Ladder size markers run in a parallel lane. The plots show the average values and variance for two repeats of the experiment. The Tables to the right of the plots indicate rate constants (k_{obs}) and amplitudes (Ampl.) for DNA products synthesized by WT and S3/G3 RT0 loop mutant G2L4 RTs with a curve fit to a first-order rate equation. Ampl. values in parentheses represent fixed amplitudes for reactions that did not reach an end point based on the average Ampl. value for those that reached a clear end point during the experiment. The red asterisk in the schematic at the bottom indicates a 5'-^{32}P-label.

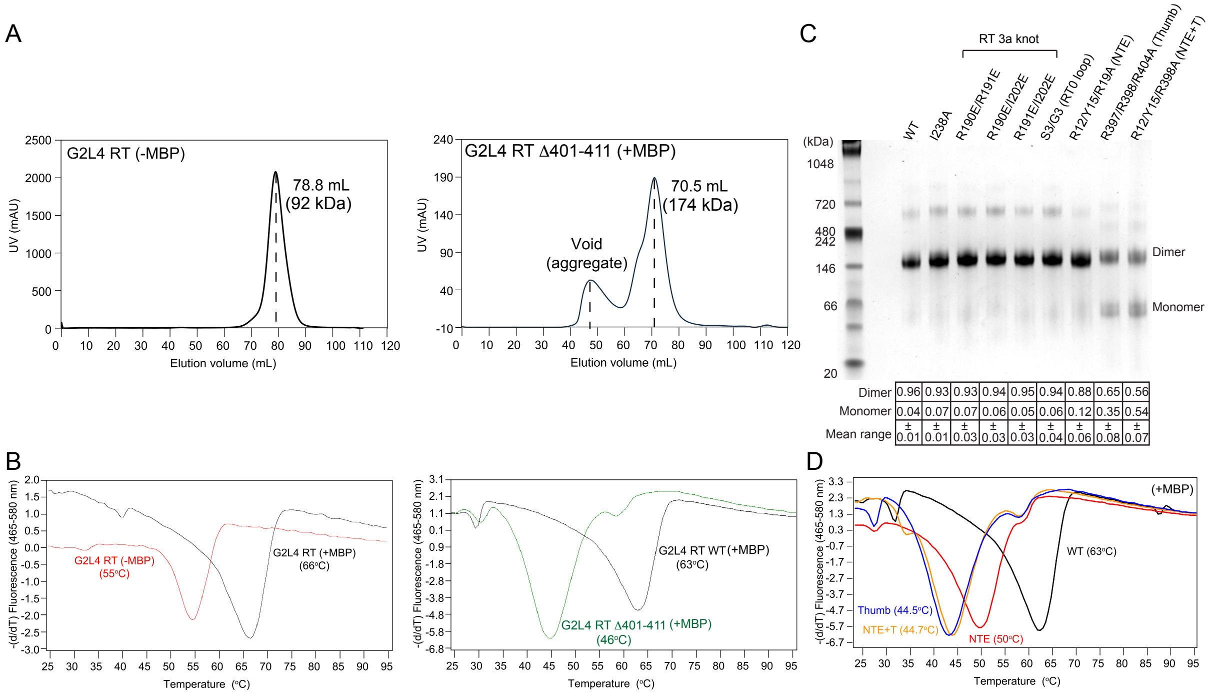

Fig. S10. Characterization of G2L4 RT dimer interface mutants. (*A*) Size-exclusion chromatography of WT G2L4 RT with a cleaved MBP tag (left panel) and a G2L4 RT C-terminal deletion mutant (Δ401-411 amino acids; right panel). The molecular weights in parenthesis were calculated based on the elution volume of the peak (dashed line) relative to the protein standard calibration graph of Fig. S1*A*. (*B*) Differential scanning fluorimetry (DSF) assays. Left panel, G2L4 RT with (black) or without (red) an MBP tag; right panel, C-terminal deletion mutant of G2L4 RT (Δ401-411 amino acids, green) versus WT G2L4 RT (black). The plots show the derivative of fluorescence intensity as a function of temperature, highlighting transitions or melting points indicative of structural changes or stability shifts. (*C*) Coomassie blue-stained non-denaturing gel for WT and mutant G2L4 RTs with an N-terminal MBP tag (1 μg/18 μL) and NativeMark Unstained Protein Standard markers run in a parallel lane. Dimer and monomer bands are labeled to the right and the mean range was calculated from three independent replicates. The fractions of monomer bands indicated below the gel were calculated by using ImageQuant TL 10.2 software. (*D*) DSF assay for dimer interface NTE mutant (R12/Y15/R19A; red); Thumb (T) domain mutant (R397/R398/R404A; blue); and NTE+T mutant (R12/Y15/R398A; orange). The plots are as described in panel *B*.

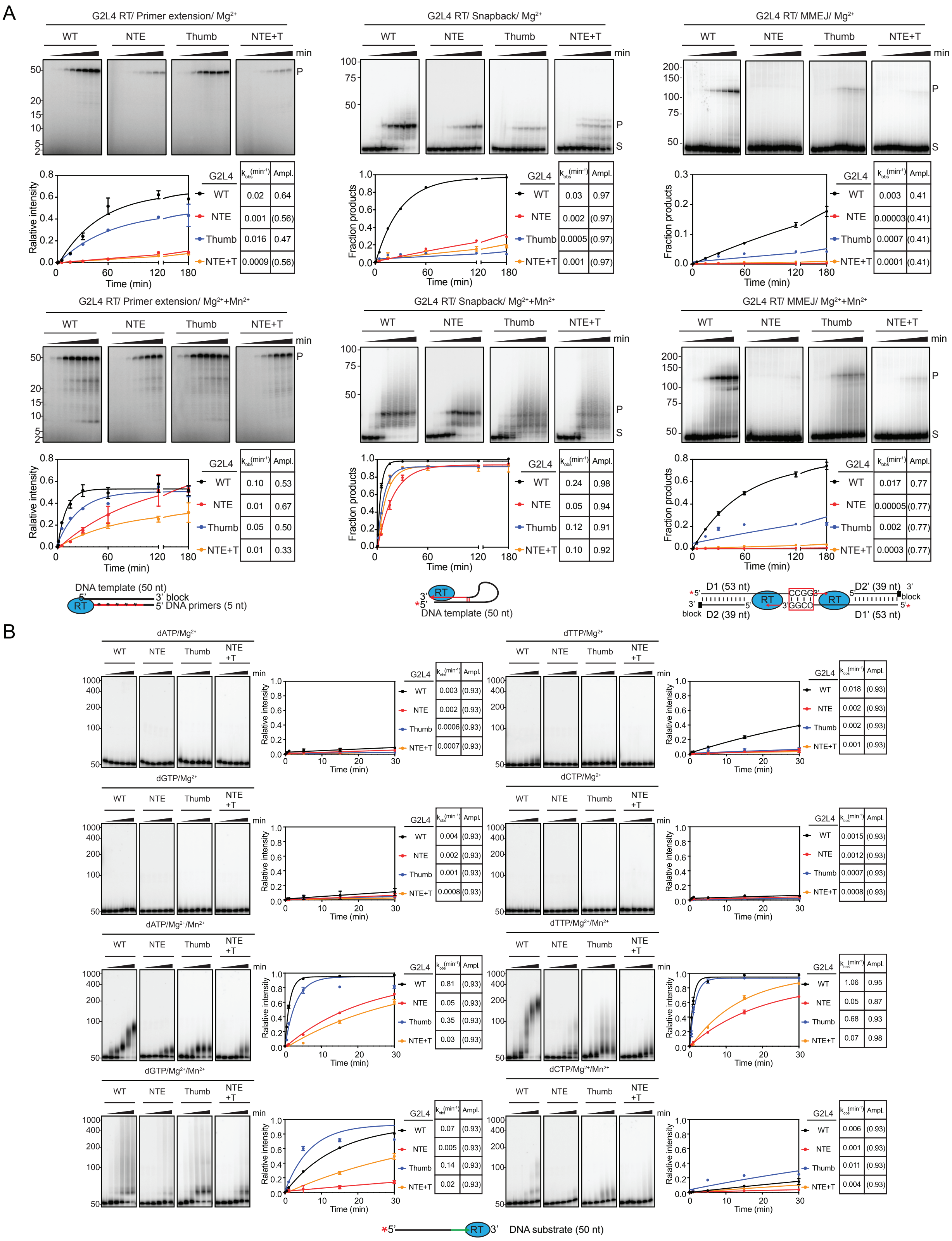

A
G2L4 RT/ Primer extension/ Mg2+
WT NTE Thumb NTE+T
min
P
G2L4 | kobs(min-1) | Ampl.
WT 0.02 0.64
NTE 0.001 (0.56)
Thumb 0.016 0.47
NTE+T 0.0009 (0.56)
Ralative intensity
Time (min)
G2L4 RT/ Snapback/ Mg2+
P
S
Fraction products
WT 0.03 0.97
NTE 0.002 (0.97)
Thumb 0.0005 (0.97)
NTE+T 0.001 (0.97)
G2L4 RT/ MMEJ/ Mg2+
WT 0.003 0.41
NTE 0.00003 (0.41)
Thumb 0.0007 (0.41)
NTE+T 0.0001 (0.41)
G2L4 RT/ Primer extension/ Mg2++Mn2+
WT 0.10 0.53
NTE 0.01 0.67
Thumb 0.05 0.50
NTE+T 0.01 0.33
G2L4 RT/ Snapback/ Mg2++Mn2+
WT 0.24 0.98
NTE 0.05 0.94
Thumb 0.12 0.91
NTE+T 0.10 0.92
G2L4 RT/ MMEJ/ Mg2++Mn2+
WT 0.017 0.77
NTE 0.00005 (0.77)
Thumb 0.002 (0.77)
NTE+T 0.0003 (0.77)
DNA template (50 nt)
5' 3' block
RT 5' DNA primers (5 nt)
3' RT 5'
DNA template (50 nt)
D1 (53 nt)
D2' (39 nt)
3' block
block D2 (39 nt)
D1' (53 nt)
CCGG
GGCC
B
dATP/Mg2+
WT 0.003 (0.93)
NTE 0.002 (0.93)
Thumb 0.0006 (0.93)
NTE+T 0.0007 (0.93)
dTTP/Mg2+
WT 0.018 (0.93)
NTE 0.002 (0.93)
Thumb 0.002 (0.93)
NTE+T 0.001 (0.93)
dGTP/Mg2+
WT 0.004 (0.93)
NTE 0.002 (0.93)
Thumb 0.001 (0.93)
NTE+T 0.0008 (0.93)
dCTP/Mg2+
WT 0.0015 (0.93)
NTE 0.0012 (0.93)
Thumb 0.0007 (0.93)
NTE+T 0.0008 (0.93)
dATP/Mg2+/Mn2+
WT 0.81 (0.93)
NTE 0.05 (0.93)
Thumb 0.35 (0.93)
NTE+T 0.03 (0.93)
dTTP/Mg2+/Mn2+
WT 1.06 0.95
NTE 0.05 0.87
Thumb 0.68 0.93
NTE+T 0.07 0.98
dGTP/Mg2+/Mn2+
WT 0.07 (0.93)
NTE 0.005 (0.93)
Thumb 0.14 (0.93)
NTE+T 0.02 (0.93)
dCTP/Mg2+/Mn2+
WT 0.006 (0.93)
NTE 0.001 (0.93)
Thumb 0.011 (0.93)
NTE+T 0.004 (0.93)
5' RT 3' DNA substrate (50 nt)

Fig. S11. Biochemical assays of G2L4 RT dimer interface mutants. (*A*) Left panels, primer extension assay; middle panels, snapback assay; right panels, MMEJ assay with10 mM Mg^{2+} or 10 mM Mg^{2+} plus 1 mM Mn^{2+}. Reactions were done with MBP-tagged dimer interface mutants as described in the legend of Fig. 2*D* and Materials and Methods. The plots show the average values and variance for two repeats of the experiment. Red asterisks in schematics indicate ^{32}P-label. (*B*) Terminal transferase assays with or without Mn^{2+} of MBP-tagged G2L4 RT dimer interface mutants using a 5'-labeled 50-nt DNA substrate, as described in Fig. S5*B*. Reactions were initiated by adding 1 mM of a single dNTP (dATP, dCTP, dGTP, and dTTP) and incubated at 37°C for times up to 30 min. The numbers to the left of the gels indicate the positions of 5'-labeled RiboRuler Low Range RNA Ladder size markers run in a parallel lane. The plots show the average values and variance for two repeats of the experiment. The Tables to the right of the plots indicate rate constants (k_{obs}) and amplitudes (Ampl.) for DNA products synthesized by WT and G2L4 RT dimer interface mutants with a curve fit to a first-order rate equation. Ampl. values in parentheses represent fixed amplitudes for reactions that did not reach an end point based on the average Ampl. value for those that reached a clear end point during the experiment. The red asterisk in the schematic at the bottom indicates a 5'-^{32}P-label.

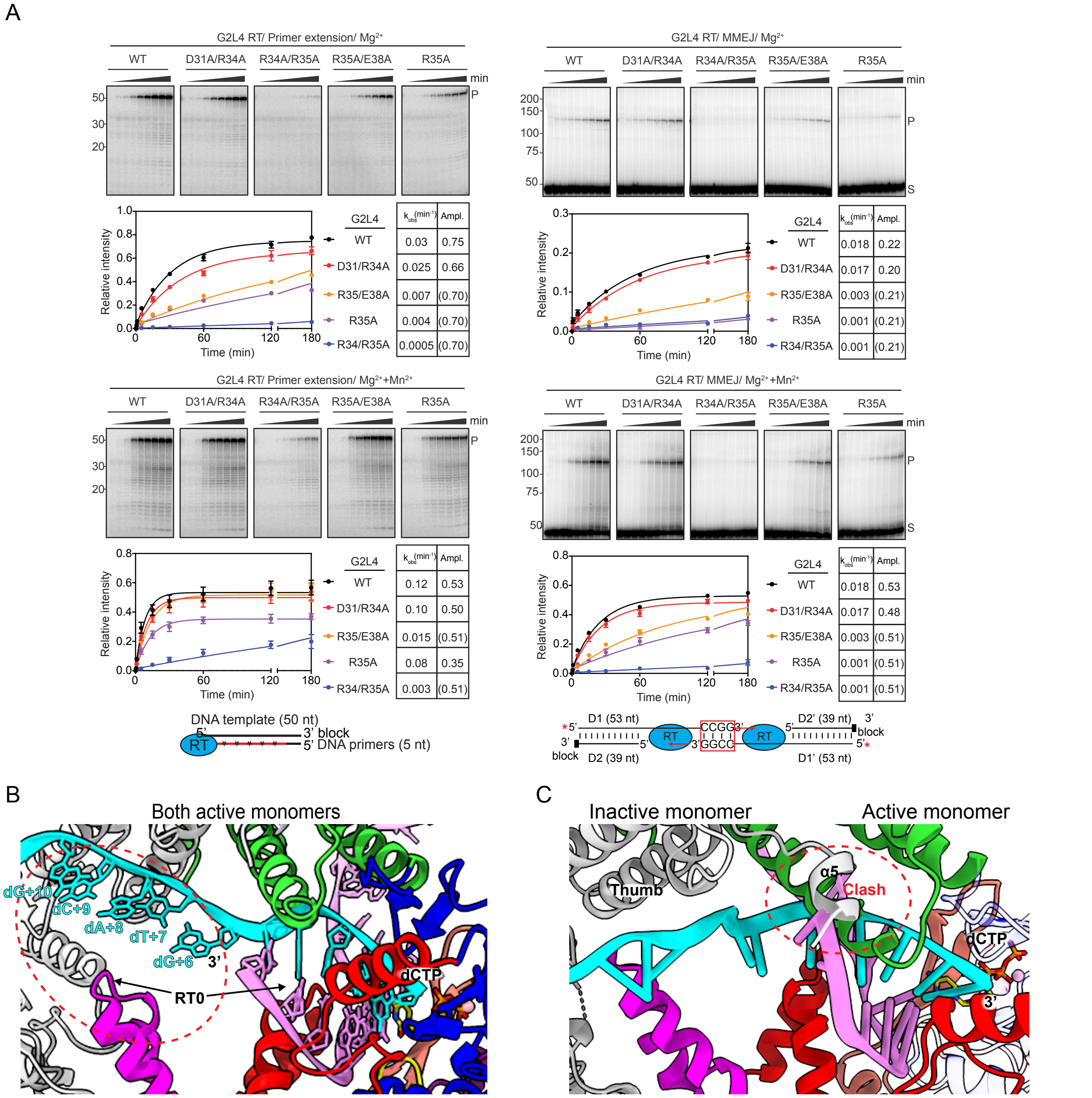

Fig. S12. Biochemical analysis and structural models supporting the suggested G2L4 RT MMEJ mechanism. (*A*) Biochemical assays of MBP-tagged G2L4 RT NTE mutants in reaction media containing 10 mM Mg^{2+} (top panels) or 10 mM Mg^{2+} plus 1 mM Mn^{2+} (bottom panels). Left panels, primer extension assays; right panels, MMEJ assays. The plots show the average values and variance for two repeats of the experiment. Ampl. values in parentheses represent fixed amplitudes for reactions that did not reach an end point based on the average Ampl. value for those that reached a clear end point during the experiment. (*B*) Model of a G2L4 RT dimer with two active monomers bound to a 5-bp annealed microhomology between the single-stranded 3' overhangs from the left side (cyan) and right side (violet) of the DSB. Protein regions are colored as in Fig. 1*A*. A dashed red circle highlights a gap between single-stranded region of the MMEJ substrate and the trailing active monomer. (*C*) Model of a G2L4 RT dimer with an active and inactive monomers bound to a 7-bp annealed microhomology between the same single-strand 3' overhangs as panel *C*. A steric clash is highlighted in a dashed red circle.

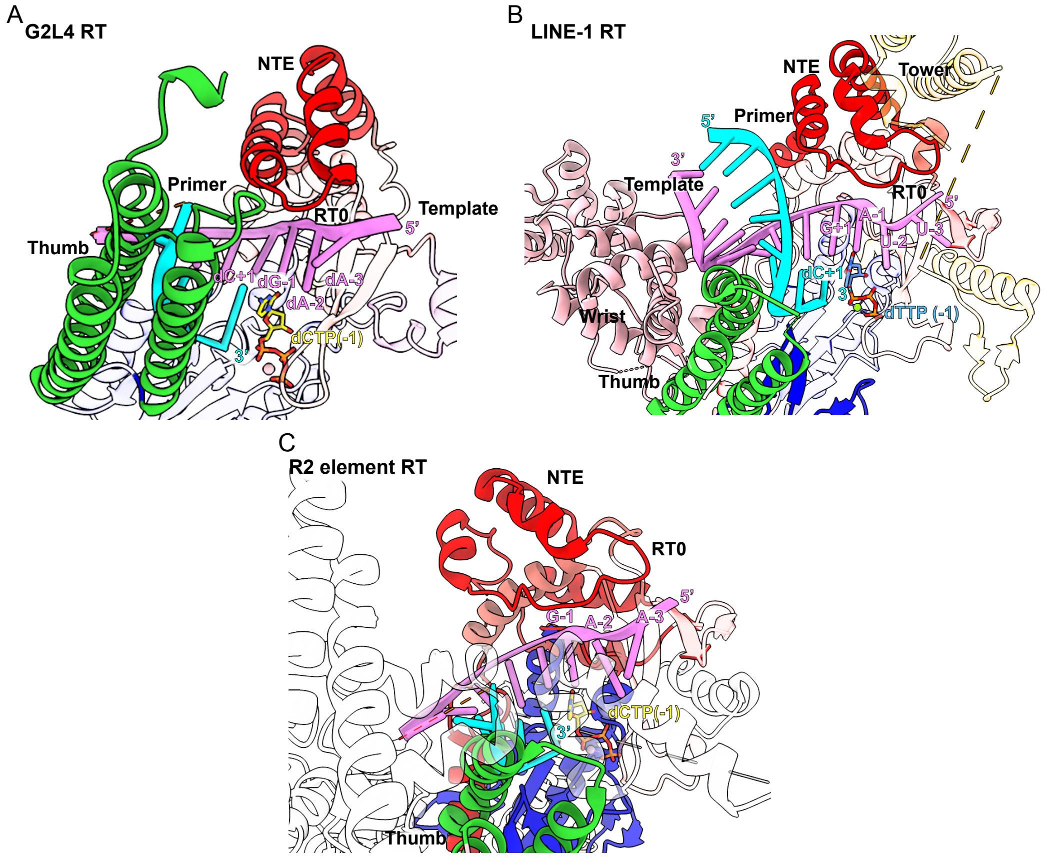

Fig. S13. Comparison of G2L4 RT NTE/RT0 loop positioned on bound snap-back DNA substrate with those in human LINE-1 and insect R2 non-LTR retroelement reverse transcriptases. *(A)* Alternate close-up view of G2L4 RT NTE/RT0 bound to a snapback DNA substrate in Fig. 4*D* (template strand, violet; primer strand, cyan), and incoming dCTP (carbons, yellow; nitrogens, blue; phosphorous, orange; oxygens, red). (*B*) Similar view of NTE/RT0 region of human LINE-1 RT (PDB: 8C8J) (26) with bound RNA template/DNA primer substrate (violet and cyan, respectively) and dTTP (stick, carbons, light blue; nitrogens, blue; phosphorus, orange; and oxygens, red). Tower (light yellow) and Wrist (pink) are additional regions of LINE-1 RT. (*C*) Similar view of the NTE/RT0 of *Bombyx mori* R2 element RT (PDB: 8GH6) (27) modeled with the same DNA substrate and dCTP as G2L4 RT as panel *A*.

Table S1. Crystallographic data collection and refinement statistics[1]

	RT apoenzyme (PDB: 9D5X)	RT snapback complex (PDB: 9D4S)	RT(Se-SAD)[2]
**Wavelength (Å)**	0.9774	1.0000	0.9774
**Resolution range (Å)**	48.75-2.61 (2.70-2.61)	47.30-2.77 (2.82-2.77)	40.78-2.90 (3.00-2.90)
**Space group**	$P2_1$	$P2_12_12_1$	$P2_1$
**Unit cell: a, b, c (Å)**	75.836, 73.227, 82.861	65.398, 99.190 ,157.276	75.077, 75.170, 83.012
**Unit cell: α, β, γ (°)**	90.00, 103.97, 90.00	90.00, 90.00, 90.00	90.00, 103.93, 90.00
**Total reflections**	182519 (17706)	192211(191330)	40010 (3973)
**Unique reflections**	26981 (2684)	26117 (2461)	20027 (1988)
**Multiplicity**	6.8 (6.6)	7.2 (6.8)	2.0 (2.0)
**Completeness (%)**	99.77 (99.89)	99.40 (99.30)	99.74 (99.45)
**Mean I/sigma**	21.11 (2.92)	11.86 (1.29)	42.65 (9.18)
**Wilson B- factor (Å²)**	64.7	56.2	66.6
**R-merge**	0.056 (0.704)	0.140 (0.836)	0.013 (0.072)
**R-meas**	0.061 (0.764)	0.150 (0.904)	0.019 (0.101)
**R-pim**	0.023 (0.296)	0.055 (0.339)	0.013 (0.072)
**$CC_{1/2}$[3]**	0.999 (0.862)	0.991 (0.782)	1.000 (0.994)
**CC***	1.000 (0.962)	0.998 (0.937)	1.000 (0.999)
**Refinement**			
**Reflections used in refinement**	26935 (2683)	26120 (2461)	
**Reflections used for R-free**	1356 (121)	1960 (188)	
**R-work**	0.2167 (0.3823)	0.2351 (0.3139)	
**R-free**	0.2430 (0.4409)	0.2763 (0.3501)	
**Number of non-hydrogen atoms[4]**	6236	7014	
**Macromolecules[5]**	6184	6949	
**Ligands**	8	58	
**Solvent**	44	7	
**Protein residues**	782	815	
**RMS (bonds) (Å)**	0.002	0.011	
**RMS (angles) (°)**	0.43	1.50	
**Ramachandran favored (%)**	98.05	97.78	
**Ramachandran allowed (%)**	1.69	1.98	
**Ramachandran outliers (%)**	0.26	0.25	
**Average B- factor (Å²)**	79.0	63.1	
**Macromolecules**	79.1	63.0	
**Ligands**	67.8	50.0	
**Solvent**	63.8	63.1	
**Molprobity score[6]**	1.09/100th percentile	1.74/100th percentile	

[1]Statistics for the highest-resolution shell are shown in parentheses.

[2]Se-SAD, seleno-methionine single-wavelength anomalous diffraction.
The outlier for both apoenzyme and complex structure is I238, a crucial active site residue. The density is clear, and the unique phi/psi angle might be related to its role in activity.

[3]$CC_{1/2}$ is the Pearson correlation coefficient for a random half of the data, the two numbers represent the lowest and highest resolution shell, respectively.

[4]Non-hydrogen atoms represent the total number of atoms included in the final refined model, encompassing protein atoms (macromolecules), ligands, and any ordered water molecules or ions, but excluding hydrogen atoms, which are typically not resolved at this resolution (2.61 Å apo and 2.77 Å complex).

[5]Macromolecules refers specifically to the protein atoms modeled in the structure, excluding solvent molecules, ligands, and other small molecules.

[6]MolProbity score is calculated by combining clashscore with rotamer and Ramachandran percentage and scaled based on X-ray resolution. The percentage is calculated with 100th percentile as the best and 0th percentile as the worst among structures of comparable resolution.

Table S2. Oligonucleotides

Use	Name	Sequence
Cloning	G2L4 RT R190E Top	5' AACATTGATGACCTTGAACGCAAGGGACTGGATGAA 3'
	G2L4 RT R191E Top	5' AACATTGATGACCTTCGCGAAAAGGGACTGGATGAA 3'
	G2L4 RT R190E/R191E Top	5’ AACATTGATGACCTTGAAGAAAAGGGACTGGATGAA 3’
	G2L4 RT R190E/R191E Bot	5’ CGCCAGATCCGTGCGGATTGCCTCTTCAAAAAGCTT 3’
	G2L4 RT I202E Top	5' GAAATCTTTCCCATTGGCGAAGAAGGGGTTGCTCAA 3'
	G2L4 RT I202E Bot	5' ATCCAGTCCCTTGCGGCGAAGGTCATCAATGTTCGC 3'
	G2L4 RT S3/G3 Top	5' TCGGCGTTTTTGGGTGGTGGTGATGAGATCCGT 3'
	G2L4 RT S3/G3 Bot	5' GGAGCGAACCTTACGCCATGCACCGTACAGGGT 3'
	G2L4 RT R19A Top	5' TCCTCGGCGTTTTTGTCTTCCAGTGATGAGATCCGTCGTGAA 3'
	G2L4 RT R19A Bot	5' GCGAACCTTTTCCCATGCACCTGCCAGGGTTTCCAGGGAGCA 3'
	G2L4 RT R397/398/404A (Thumb) Top	5' ACAGTAGCAGCACGTGTATTAGGCGTGGCATTGCTTATT 3'
	G2L4 RT R397/398/404A (Thumb) Bot	5' ACTGTCGCTGTTGGCAAGAATGGCAATTTTTTC 3'
	G2L4 RT R12/Y15A Top	5' CGTAAGGTTCGCTCCTCGGCGTTTTTGTCTTCC 3'
	G2L4 RT R12/Y15A Bot	5' CCATGCACCCGCCAGGGTCGCCAGGGAGCAAAT 3'
	G2L4 RT R398A Top	5' GACAGTACAGTACGTGCCCGTGTATTAGGCGTG 3'
	G2L4 RT R398A Bot	5' GCTGTTGGCAAGAATGGCAATTTTTTCCGCTTC 3'
	G2L4 RT C-term deletion (401-411) Top	5' TGAGGATCCGAATTCCCTGCAGGTA 3'
	G2L4 RT C-term deletion (401-411) Bot	5' TACACGACGACGTACTGTACTGTCGC 3'
	G2L4 RT D31A/R34A Top	5’ ATCGCGCGTGAAGCAGAGGAATTTGAGAG 3’
	G2L4 RT D31A/R34A Bot	5’ CTCCGCACTGGAAGACAAAAACGCCGAG 3’
	G2L4 RT R34A/R35A Top	5’ TGATGAGATCGCAGCAGAAGCAGAGGAATTTGAGAGTCGTTTGC 3’
	G2L4 RT R34A/R35A Bot	5’ CTGGAAGACAAAAACGCCGAGG 3’
	G2L4 RT R35A/E38A Top	5’ GCAGCAGAATTTGAGAGTCGTTTGCCCGAC 3’
	G2L4 RT R35A/E38A Bot	5’ TTCCGCACGGATCTCATCACTGGAAGAC 3’
	G2L4 RT R35A Top	5’ TGAGATCCGTGCGGAAGCAGAGGAATTTG 3’
	G2L4 RT R35A Bot	5’ TCACTGGAAGACAAAAACGCCG 3’
Snapback/ Terminal transferase assays	50-nt DNA	5' GCAATAATCTATACAATACAACACATACAAACAAATTCTTAAGGTCCCAA 3'
Primer extension assays	50-nt DNA 3' block (Inverted dT at the 3'-end)	5' GCAATAATCTATACAATACAACACATACAAACAAATTCTTAAGGTCCCAA/InvdT/ 3'
	5-nt DNA primer	5' TTGGG 3'
MMEJ assays	53-nt DNA 4 microhomology (CCGG) Top	5' CCCTGTACAGTAAGAGCCTACTCATGGATCCTCCTTGTGATGTAAGGTCCCGG 3'
	39-nt DNA 3' block (Inverted dT at the 3'-end) Bot	5' ACAAGGAGGATCCATGAGTAGGCTCTTACTGTACAGGG/ InvdT / 3'
Snapback substrate for X-ray crystallography	15-nt snapback DNA	5’ AAGCGGTTAACCCAA 3’

Table S3. Recombinant Plasmids

Name	Source
pMal-G2L4 RT WT	Park et al., 2022 (1)
pMal-G2L4 RT I238A	Park et al., 2022 (1)
pMal-G2L4 RT R190E/R191E	This Study
pMal-G2L4 RT R190E/I202E	This Study
pMal-G2L4 RT R191E/I202E	This Study
pMal-G2L4 RT S3/G3	This Study
pMal-G2L4 RT R12/Y15/R19A (NTE)	This Study
pMal-G2L4 RT R397/R398/R404A (Thumb)	This Study
pMal-G2L4 RT R12/Y15/R398A (NTE+T)	This Study
pMal-G2L4 RT D31A/D34A	This Study
pMal-G2L4 RT D34A/D35A	This Study
pMal-G2L4 RT D35A/D38A	This Study
pMal-G2L4 RT D35A	This Study

SI References

1. S. K. Park, G. Mohr, J. Yao, R. Russell, A. M. Lambowitz, Group II intron-like reverse transcriptases function in double-strand break repair. *Cell* **185**, 3671-3688.e23 (2022).

2. G. E. Crooks, G. Hon, J.-M. Chandonia, S. E. Brenner, WebLogo: A Sequence Logo Generator: Figure 1. *Genome Res.* **14**, 1188–1190 (2004).

3. S. Altschul, Gapped BLAST and PSI-BLAST: a new generation of protein database search programs. *Nucleic Acids Research* **25**, 3389–3402 (1997).

4. A. M. Waterhouse, J. B. Procter, D. M. A. Martin, M. Clamp, G. J. Barton, Jalview Version 2--a multiple sequence alignment editor and analysis workbench. *Bioinformatics* **25**, 1189–1191 (2009).

5. J. D. Thompson, D. G. Higgins, T. J. Gibson, CLUSTAL W: improving the sensitivity of progressive multiple sequence alignment through sequence weighting, position-specific gap penalties and weight matrix choice. *Nucleic Acids Res* **22**, 4673–4680 (1994).

6. S. Zimmerly, L. Wu, An Unexplored Diversity of Reverse Transcriptases in Bacteria. *Microbiol Spectr* **3**, 3.2.13 (2015).

7. C. Notredame, D. G. Higgins, J. Heringa, T-Coffee: A novel method for fast and accurate multiple sequence alignment. *J Mol Biol* **302**, 205–217 (2000).

8. W. Kabsch, XDS. *Acta Crystallogr D Biol Crystallogr* **66**, 125–132 (2010).

9. P. R. Evans, G. N. Murshudov, How good are my data and what is the resolution? *Acta Crystallogr D Biol Crystallogr* **69**, 1204–1214 (2013).

10. P. D. Adams, *et al.*, PHENIX: a comprehensive Python-based system for macromolecular structure solution. *Acta Crystallogr D Biol Crystallogr* **66**, 213–221 (2010).

11. P. Emsley, B. Lohkamp, W. G. Scott, K. Cowtan, Features and development of Coot. *Acta Crystallogr D Biol Crystallogr* **66**, 486–501 (2010).

12. G. N. Murshudov, *et al.*, REFMAC5 for the refinement of macromolecular crystal structures. *Acta Crystallogr D Biol Crystallogr* **67**, 355–367 (2011).

13. P. Hehenberger, D. Bradley, *Mechatronic futures: challenges and solutions for mechatronic systems and their designers* (Springer International Publishing, 2016).

14. Z. Otwinowski, W. Minor, Processing of X-ray diffraction data collected in oscillation mode. *Methods Enzymol* **276**, 307–326 (1997).

15. P. Emsley, K. Cowtan, Coot: model-building tools for molecular graphics. *Acta Crystallogr D Biol Crystallogr* **60**, 2126–2132 (2004).

16. C. J. Williams, *et al.*, MolProbity: More and better reference data for improved all-atom structure validation. *Protein Sci* **27**, 293–315 (2018).

17. E. F. Pettersen, *et al.*, UCSF ChimeraX: Structure visualization for researchers, educators, and developers. *Protein Sci* **30**, 70–82 (2021).

18. D. Liebschner, *et al.*, Macromolecular structure determination using X-rays, neutrons and electrons: recent developments in Phenix. *Acta Crystallogr D Struct Biol* **75**, 861–877 (2019).

19. C. A. Schneider, W. S. Rasband, K. W. Eliceiri, NIH Image to ImageJ: 25 years of image analysis. *Nat Methods* **9**, 671–675 (2012).

20. K. Büttner, S. Nehring, K.-P. Hopfner, Structural basis for DNA duplex separation by a superfamily-2 helicase. *Nat Struct Mol Biol* **14**, 647–652 (2007).

21. G. M. Sastry, M. Adzhigirey, T. Day, R. Annabhimoju, W. Sherman, Protein and ligand preparation: parameters, protocols, and influence on virtual screening enrichments. *J Comput Aided Mol Des* **27**, 221–234 (2013).

22. J. Kyte, R. F. Doolittle, A simple method for displaying the hydropathic character of a protein. *J Mol Biol* **157**, 105–132 (1982).

23. C. Zhao, A. M. Pyle, Crystal structures of a group II intron maturase reveal a missing link in spliceosome evolution. *Nat Struct Mol Biol* **23**, 558–565 (2016).

24. J. L. Stamos, A. M. Lentzsch, A. M. Lambowitz, Structure of a Thermostable Group II Intron Reverse Transcriptase with Template-Primer and Its Functional and Evolutionary Implications. *Mol Cell* **68**, 926-939.e4 (2017).

25. P. Y. Chou, G. D. Fasman, Empirical predictions of protein conformation. *Annu Rev Biochem* **47**, 251–276 (1978).

26. E. T. Baldwin, *et al.*, Structures, functions and adaptations of the human LINE-1 ORF2 protein. *Nature* **626**, 194–206 (2024).

27. M. E. Wilkinson, C. J. Frangieh, R. K. Macrae, F. Zhang, Structure of the R2 non-LTR retrotransposon initiating target-primed reverse transcription. *Science* **380**, 301–308 (2023).
